# Tubocapsenolide A Overcomes Oxaliplatin Resistance in Colorectal Cancer by Targeting PLK1 to Impair CEP55–ESCRT‐Associated Vesicular Damage Clearance

**DOI:** 10.1002/advs.78310

**Published:** 2026-10-18

**Authors:** Yueying Yang, Jing Liang, Lu Wang, Hongyan Zhang, Zhiruo Zhang, Yang Liu, Rui Zhu, Wen Xu, Shaohua Xu, Dejuan Sun, Ke Xiang, Yali Wang, Zhimin Fan, Zheng Wang, Hua Li, Lixia Chen

**Affiliations:** ^1^ Wuya College of Innovation Shenyang Pharmaceutical University Shenyang People's Republic of China; ^2^ Institute of Digital Materia Medica and Structural Pharmacology, Fujian Key Laboratory of Chinese Materia Medica, College of Pharmacy Fujian University of Traditional Chinese Medicine Fuzhou People's Republic of China; ^3^ Jiangsu Clinical Innovation Center for Anorectal Diseases of T.C.M Nanjing Hospital of Chinese Medicine Nanjing University of Chinese Medicine Nanjing People's Republic of China; ^4^ Research Center for Tissue Engineering and Regenerative Medicine Union Hospital Tongji Medical College Huazhong University of Science and Technology Wuhan People's Republic of China; ^5^ Department of Gastrointestinal Surgery Union Hospital Tongji Medical College Huazhong University of Science and Technology Wuhan People's Republic of China

**Keywords:** colorectal cancer, ESCRT, platinum resistance, PLK1, withanolide

## Abstract

Oxaliplatin (L‐OHP) is a first‐line chemotherapeutic agent for advanced colorectal cancer (CRC) and postoperative adjuvant therapy. However, acquired L‐OHP resistance remains a clinical obstacle. Here, we identify Tubocapsenolide A (TA), a natural withanolide isolated from *Tubocapsicum anomalum*, as a promising antitumor agent that suppresses CRC growth and sensitizes L‐OHP‐resistant CRC to L‐OHP. Using a PROTAC‐based target deconvolution strategy, we identify PLK1 as a direct molecular target of TA. PLK1 is elevated in L‐OHP‐resistant CRC and is associated with reduced patient sensitivity to L‐OHP. Mechanistically, elevated PLK1 promotes CEP55 Ser436 phosphorylation and facilitates ESCRT recruitment to nuclear and mitochondrial budding sites. This process facilitates the clearance of L‐OHP‐induced damaged components through nuclear budding and TOM20^−^/PDH^+^ mitochondrial‐derived vesicles, thereby contributing to organelle homeostasis and cell survival. By inhibiting PLK1, TA interferes with this vesicle‐associated damage clearance process, leading to intracellular accumulation of damaged components. This damage accumulation is accompanied by nucleolar stress and mitochondrial dysfunction, ultimately contributing to cell death. Collectively, our findings identify a previously unappreciated role of the PLK1–CEP55–ESCRT axis in vesicle‐associated damage clearance during L‐OHP resistance and support this axis as a potential therapeutic target. TA represents a promising lead compound for overcoming L‐OHP resistance in CRC.

## Graphical abstract

Oxaliplatin‐resistant colorectal cancer cells utilize a previously unappreciated PLK1‐CEP55‐ESCRT‐driven vesicular extrusion mechanism to clear L‐OHP‐induced cellular damage. The natural withanolide Tubocapsenolide A directly targets and inhibits PLK1 to disrupt this specific damage‐clearance machinery. This blockade induces severe nucleolar stress and mitochondrial dysfunction, offering a promising therapeutic strategy for chemorefractory colorectal cancer.

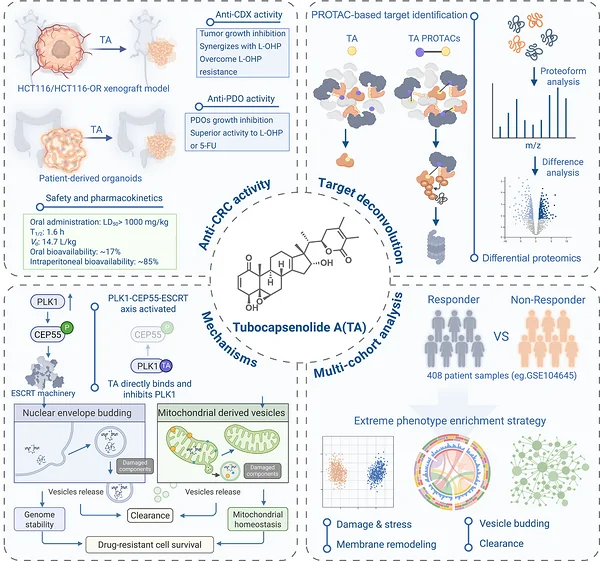

**Abbreviations**
4‐HNE: 4‐hydroxynonenal
53BP1: p53‐binding protein 1
5‐FU: 5‐fluorouracil
8‐OHdG: 8‐hydroxy‐2’‐deoxyguanosine
ALIX: ALG‐2 (Apoptosis‐linked Gene 2)‐interacting Protein X
CD: Kinase Domain
CDC7: Cell Division Cycle 7
CDKN1A: Cyclin Dependent Kinase Inhibitor 1A
CEP55: Centrosomal Protein of 55 kDa
CETSA: Cellular Thermal Shift Assay
COREAD: Colorectal Adenocarcinoma
CRC: Colorectal Cancer
DARTS: Drug Affinity Responsive Target Stability
ESCRT: Endosomal Sorting Complexes Required for Transport
FTL: Ferritin Light Chain
GPX4: Glutathione Peroxidase 4
GSH: Glutathione
HMOX1: Heme Oxygenase 1
KIF23: Kinesin family Member 23
Lamin A/C: Lamin A and Lamin C
L‐OHP: Oxaliplatin
MDA: Malondialdehyde
MDV: Mitochondrial‐derived Vesicle
MFI: Mean Fluorescence Intensity
MOA: Mechanism of Action
MST: Microscale Thermophoresis
MYC: MYC Proto‐oncogene Protein
Nrf2: Nuclear Factor Erythroid 2‐related Factor 2
OPLS‐DA: Orthogonal Partial Least Squares Discriminant Analysis
PBD: Polo‐box Domain
PDH: Pyruvate Dehydrogenase (Lipoamide) Alpha 1
PDO: Patient‐derived Organoid
PLK1: Serine/Threonine‐protein Kinase PLK1
PROTAC: Proteolysis‐targeting Chimera
ROS: Reactive Oxygen Species
SOD2: Superoxide Dismutase 2
SPR: Surface Plasmon Resonance
TA: Tubocapsenolide A
TCGA: The Cancer Genome Atlas
TEM: Transmission Electron Microscopy
TOM20: Mitochondrial Import Receptor Subunit TOM20 Homolog
TSG101: Tumor Susceptibility Gene 101 Protein
γ‐H2A.X: Gamma‐H2A histone family member X

## 1 Introduction

Colorectal cancer (CRC) is the third most commonly diagnosed malignancy and the second leading cause of cancer‐related mortality worldwide, accounting for approximately 900 000 deaths annually [1]. Chemotherapy is an indispensable and crucial strategy for CRC treatment [2]. Oxaliplatin (L‐OHP), a third‐generation platinum‐based antitumor agent, is primarily utilized as first‐line therapy for advanced CRC and as adjuvant treatment following complete resection of primary tumors [3]. Despite its initial clinical efficacy, acquired resistance to L‐OHP develops in nearly 50% of patients with stage II/III CRC, representing a major clinical challenge [4]. Therefore, it is imperative to elucidate the complex molecular mechanisms underlying L‐OHP resistance and to identify novel, effective therapeutic strategies to overcome this clinical challenge.

Current understanding of the mechanisms underlying platinum‐based drug resistance primarily focuses on restricting intracellular drug accumulation, enhancing DNA damage repair, and activating anti‐apoptotic signaling pathways [5, 6, 7]. In response to chemotherapy‐induced genotoxic and mitochondrial stress, autophagy can contribute to cellular quality control and promote cancer cell survival and chemoresistance [8, 9]. Macroautophagy can mediate bulk sequestration and, in the context of organelle quality control, turnover of substantial portions or entire organelles. Such pathways may be less suited for the rapid and spatially selective handling of localized suborganelle damage [10, 11]. How L‐OHP‐resistant CRC cells rapidly handle localized damage while preserving the integrity of surrounding cellular structures remains poorly understood. Recent studies suggest that membrane‐remodeling processes, including nuclear budding and the formation of mitochondrial‐derived vesicles (MDVs), may contribute to the selective handling of damaged cellular components and maintenance of cellular homeostasis [12, 13, 14]. The endosomal sorting complexes required for transport (ESCRT) machinery has emerged as a versatile membrane‐remodeling system that mediates membrane scission and repair across diverse cellular compartments, with recent studies further implicating ESCRT‐III in nuclear and micronuclear envelope remodeling and organelle homeostasis [12, 13, 15]. However, whether chemoresistant CRC cells engage these membrane‐remodeling processes to handle L‐OHP‐induced damage remains unknown.

Leveraging their chemical structural diversity and the specific target affinities conferred by long‐term evolution, natural products serve as an abundant resource for discovering lead compounds with novel mechanism of action (MOA), and a valuable source for drug discovery [16]. Withanolides, a class of C28 steroidal lactones primarily isolated from plants of the Solanaceae family, have attracted considerable attention in medicinal chemistry [17, 18]. As a representative member, Withaferin A from the famous Indian medicinal plant *Withania somnifera* (Ashwagandha) is currently under clinical investigation for advanced osteosarcoma [18]. As a core research focus of our group, we have previously characterized a series of promising lead candidates from this class that demonstrate significant anti‐tumor properties [17, 18, 19, 20, 21]. Among these, Tubocapsenolide A (TA), a major withanolide isolated from *Tubocapsicum anomalum*, has shown potent activity against CRC. The present study aims to elucidate the direct targets and the underlying mechanisms of TA, thereby providing candidates and therapeutic strategies for refractory CRC.

Here, we show that L‐OHP‐resistant CRC cells engage in a PLK1–CEP55–ESCRT‐associated damage clearance program. Specifically, these resistant cells exhibit elevated PLK1 expression, which promotes CEP55 Ser436 phosphorylation and facilitates ESCRT recruitment to nuclear and mitochondrial budding sites. This process is associated with increased γ‐H2A.X^+^ nuclear budding and TOM20^−^/PDH^+^ MDVs, potentially facilitating the removal of damaged nuclear and mitochondrial components, while preserving the overall structural and functional integrity of parental organelles. Importantly, we identify TA as a direct PLK1‐targeting compound that interferes with the CEP55‐ESCRT‐associated vesicular scission process, impairing nuclear bud abscission and promoting the intra‐nuclear accumulation of damaged DNA. Simultaneously, TA suppresses MDV formation and release, accompanied by mitochondrial dysfunction and oxidative stress that ultimately contribute to cell death in L‐OHP‐resistant cells. Collectively, these findings identify a previously unrecognized role of the PLK1–CEP55–ESCRT axis in L‐OHP resistance and support TA as a potential lead compound for overcoming chemoresistance in CRC.

## 2 Results

### 2.1 TA Exhibits Antitumor Efficacy Against CRC With Favorable Tolerability

TA showed potent, dose‐dependent growth inhibition across a panel of CRC cell lines, with half‐maximal inhibitory concentrations (IC_50_) values ranging from 0.47 to 4.02 µm. HCT116 and DLD‐1 were the most sensitive lines (Figure S1A). To evaluate the in vivo anti‐CRC efficacy of TA, we tested TA in HCT116 and DLD‐1 xenograft models. The results showed that intragastric (i.g.) administration of TA at doses of 10–40 mg/kg significantly suppressed tumor progression in a dose‐dependent manner without causing significant body weight loss (Figure 1A–C and Figure S1B–D). While the positive control 10 mg/kg L‐OHP exhibited comparable tumor‐inhibitory effects in the HCT116 model, it caused substantial body weight loss in the mice, whereas TA treatment was well tolerated under the tested conditions (Figure 1D).

**FIGURE 1:**
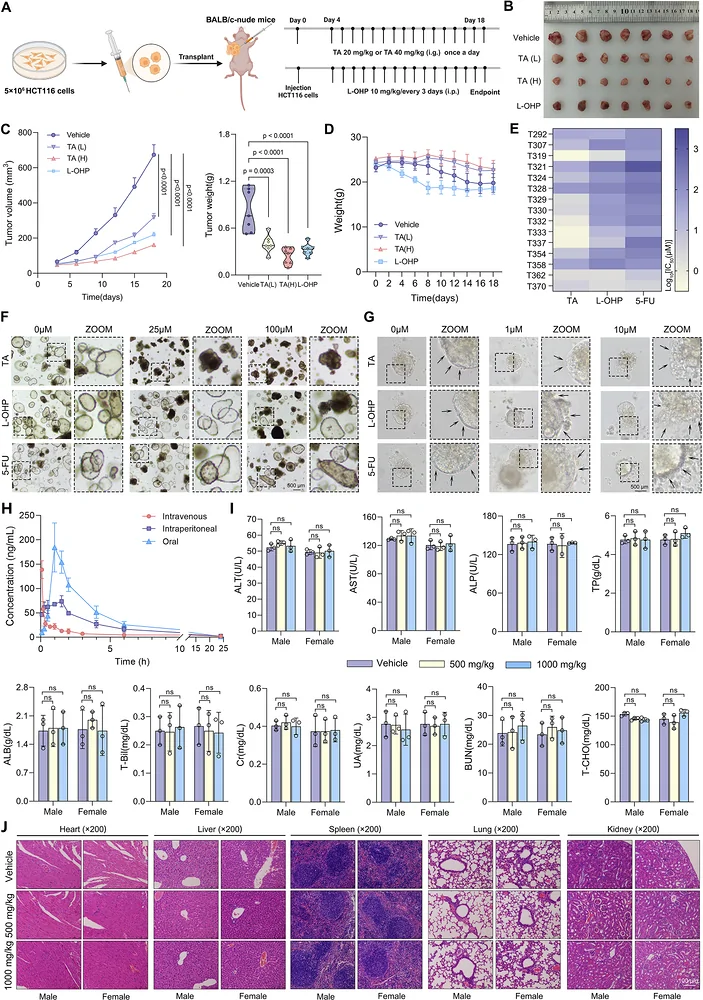
TA potently inhibits CRC growth with favorable tolerability. (A) Schematic diagram illustrating the establishment of the HCT116 subcutaneous xenograft mouse model and the therapeutic regimen, in which BALB/c‐nude mice were treated with vehicle, TA (L) (20 mg/kg i.g.) or TA (H) (40 mg/kg i.g.) once daily for 18 days, or L‐OHP (10 mg/kg i.p.) every three days. (B) Representative photographs of the excised xenograft tumors from mice treated with Vehicle, TA, or L‐OHP (*n* = 7). (C) Tumor growth curves depicting tumor volume over time (left) and box plots of final tumor weights (right) across the indicated treatment groups. (D) Dynamic monitoring of mouse body weights throughout the 18‐day treatment period. Tumor volume is presented as mean ± s.e.m. (*n* = 7). Tumor weight and mouse body weight are presented as mean ± s.d. (*n* = 7). (E) Heatmap demonstrating the IC_50_ values (Log_10_ transformed) of TA, L‐OHP, and 5‐FU across a panel of PDOs. (F, G) Representative bright‐field microscopic images illustrating the dose‐dependent morphological disintegration and viability loss of PDOs following treatment with TA, L‐OHP, or 5‐FU at indicated concentrations. Scale bar, 500 µm. (H) Plasma concentration‐time profiles of TA in Wistar rats over a 24 h period following a single intravenous (i.v., 0.5 mg/kg), intraperitoneal (i.p., 2.5 mg/kg), or intragastric (i.g., 25 mg/kg) administration. *n* = 7 rats per group. (I) Comprehensive serum biochemical analysis of hepatic and renal function markers (ALT, AST, ALP, TP, ALB, T‐Bil, Cr, UA, BUN, T‐CHO) in healthy male and female BALB/c mice following intragastric administration of high‐dose TA (500 mg/kg and 1000 mg/kg). *n* = 3 mice per group. Data in H and I are shown as the mean ± s.d. Statistical analysis was performed using one‐way ANOVA followed by Tukey's multiple comparisons test. Exact p‐values are indicated in the figures. ns, not significant. (J) Representative H&E staining images of major vital organs (heart, liver, spleen, lung, and kidney) from BALB/c mice in the acute toxicity study. Magnification, ×200. Scale bar, 100 µm.

To further evaluate the clinical relevance of TA, we utilized patient‐derived organoids (PDOs) established from primary CRC tissues. IC_50_ heatmap analysis revealed that PDOs exhibited higher sensitivity to TA compared with the conventional chemotherapeutic agents L‐OHP and 5‐fluorouracil (5‐FU), against which most organoids showed considerable resistance (Figure 1E). We next compared the morphological effects of TA, L‐OHP, and 5‐FU in both 2D monolayer cultures and 3D organoids. In 2D cultures, TA treatment rapidly transformed the semi‐transparent cystic structures into dark, condensed, and shrunken apoptotic aggregates (Figure 1F). In 3D tumor organoids, vehicle‐treated controls maintained compact and well‐organized spherical morphology, whereas TA induced pronounced disruption of organoid edges, extensive fragmentation, and eventual complete disintegration (Figure 1G). Notably, TA exerted stronger disruptive effects than L‐OHP or 5‐FU at equivalent concentrations.

Given the critical importance of safety in drug development, we evaluated the pharmacokinetic (PK) properties and acute toxicity of TA. PK studies revealed that TA undergoes relatively rapid systemic elimination following intravenous injection (T_1/2_ ≈ 1.6 h), accompanied by extensive tissue distribution (*V*_d_ = 14.7 L/kg). Interestingly, oral administration resulted in a prolonged apparent half‐life (∼ 4.0 h), indicating a typical flip‐flop pharmacokinetic profile where delayed gastrointestinal absorption acts as the rate‐limiting step. TA displayed a low absolute oral bioavailability (∼ 17%) compared with its high intraperitoneal bioavailability (∼ 85%), suggesting that the low oral exposure may reflect limited absorption and/or presystemic metabolism (Figure 1H and Table S1). In the acute toxicity assessment, sequential oral dose escalation of TA up to 1000 mg/kg resulted in no mortality or apparent behavioral abnormalities in either male or female mice, with no significant alterations in visceral organ indices (Figure S1E). Moreover, serum biochemical analyses in both healthy and tumor‐bearing mice revealed no significant fluctuations in key markers indicative of hepatic (ALT, AST, T‐Bil), renal (Cr, BUN, UA, UREA), and cardiac (CK, CK‐MB) functions (Figure 1I and Figure S1F). Histopathological examination by H&E staining of major vital organs from both the acute toxicity cohort and the xenograft model exhibited completely intact tissue architectures without any signs of lesions, necrosis, or inflammatory infiltration (Figure 1J and Figure S1G,H). Collectively, these results support the therapeutic potential of TA for CRC, with favorable tolerability in the tested preclinical models.

### 2.2 Identification of PLK1 as the Direct and Functional Target of TA

Given the well‐documented polypharmacology and typically moderate‐to‐weak binding affinities of natural products, we previously developed a Proteolysis‐Targeting Chimera (PROTAC)‐based target identification strategy coupled with TMT quantitative proteomics [22, 23]. This event‐driven, catalytically cyclic approach enables efficient degradation of low‐affinity targets through ternary complex formation [24, 25]. This approach has been successfully applied to several natural products [26, 27, 28]. Here, we combined it with TMT quantitative proteomics to systematically and unbiasedly deconvolute the direct targets of TA. A series of TA‐derived PROTACs incorporating different E3 ligase ligands was synthesized (Figure 2A). Among them, TA‐N and TA‐P displayed potent antiproliferative activity in HCT116 cells, with IC_50_ values of 0.490 and 0.512 µm, respectively, comparable to that of the parent compound TA (0.472 µm), whereas TA‐V and TA‐B were less potent (Figure S2A). Therefore, TA‐N and TA‐P were selected for subsequent target deconvolution. TMT‐based quantitative proteomics following TA‐N treatment identified Polo‐like kinase 1 (PLK1) as the most significantly downregulated protein, identifying it as the direct functional target (Figure 2B,C). Immunoblotting confirmed that TA‐N selectively and time‐dependently degraded PLK1 without affecting other PLK family members (PLK2, PLK3, or PLK4), whereas treatment with TA or Nutlin‐3 alone did not induce PLK1 degradation (Figure S2B–E). Both TA and Nutlin‐3 competitively inhibited TA‐N‐induced PLK1 degradation, and the proteasome inhibitor MG132 similarly blocked this degradation (Figure S2F). Moreover, TA‐N promoted the ubiquitination of PLK1 in a dose‐ and time‐dependent manner, an effect that was reversed by TA or Nutlin‐3 (Figure S2G,H). Collectively, these findings demonstrate that TA‐N specifically mediates PLK1 degradation via activation of the ubiquitin‐proteasome system, establishing PLK1 as a direct molecular target of TA.

**FIGURE 2:**
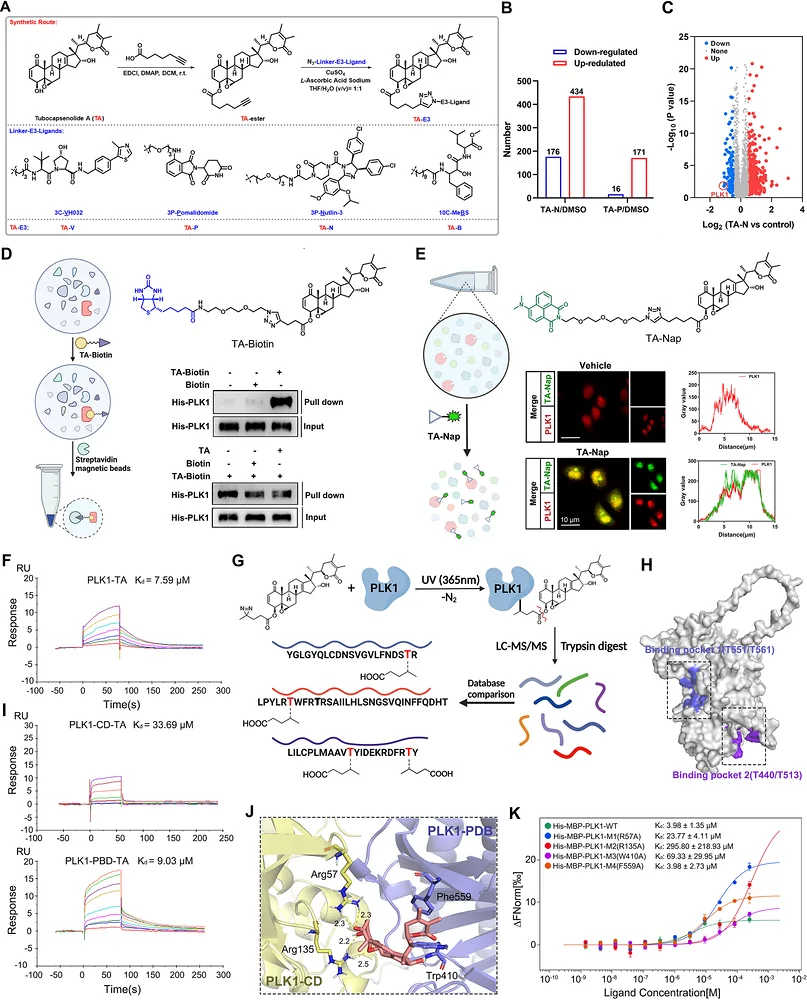
Identification and validation of PLK1 as the direct target of TA. (A) Synthetic route and chemical structures of TA‐derived PROTACs incorporating different E3 ligase ligands. (B) Numbers of differentially expressed proteins in the TA‐N/DMSO and TA‐P/DMSO experimental settings, respectively. (C) Volcano plot of quantitative proteomics data (TA‐N vs control), with down‐regulated (blue) and up‐regulated (red) proteins plotted based on fold change vs. ‐Log_10_(p‐value). (D) Schematic representation of the pull‐down assay using TA‐Biotin. Recombinant His‐PLK1 was incubated with 5 µm TA‐Biotin for 3 h, followed by capture with streptavidin‐conjugated magnetic beads. For competitive experiments, recombinant His‐PLK1 was pre‐incubated with 5 µm free TA or biotin for 1 h prior to the addition of 5 µm TA‐Biotin for another 3 h. (E) Colocalization analysis of PLK1 and TA‐Nap in HCT116 cells. Cells were incubated for 3 h with vehicle or 5 µm TA‐Nap (green), followed by immunostaining with an anti‐PLK1 antibody (red). Fluorescence intensity profiles of TA‐Nap and PLK1 along the indicated lines were generated using the Plot Profile function in ImageJ and plotted as gray value vs. distance. Scale bar, 10 µm. (F, I) SPR sensorgrams evaluating the K_d_ of TA to full‐length PLK1, PLK1‐CD, and PLK1‐PBD. (G) Schematic illustration of the photoaffinity labeling (PAL) coupled with LC‐MS/MS strategy used to map the precise binding sites of TA on PLK1. Recombinant PLK1 was incubated with a TA‐DA probe and covalently cross‐linked via UV irradiation (365 nm). Subsequent trypsin digestion and tandem mass spectrometry analysis identified the specific modified peptides. (H) Mapping of the two potential TA‐binding pockets on the AlphaFold‐predicted 3D surface model of PLK1 (AF‐P53350‐F1). Guided by the PAL‐LC‐MS/MS results, Binding pocket 1 (blue) and Binding pocket 2 (purple) are highlighted, encompassing the identified cross‐linked residues T551/T561 and T440/T513, respectively. (J) Structural model of TA (pink sticks) docked into the predicted binding pocket 1 at the interface between PLK1‐CD and PLK1‐PBD. Key interacting residues, Arg57, Arg135, Trp410, and Phe559, are shown as sticks, with predicted hydrogen bonds and distances (in Å) indicated by yellow dashed lines. (K) MST analysis of TA binding to wild‐type His‐MBP‐PLK1 and the indicated point mutants, showing binding curves and K_d_. Data are presented as mean ± s.d. (*n* = 3).

To further validate the direct interaction between TA and PLK1, we performed a series of biophysical assays. Affinity pull‐down assays utilizing TA‐Biotin successfully enriched recombinant His‐PLK1, and this enrichment was competitively abrogated by excess free TA (Figure 2D and Scheme S1). Additionally, a fluorescent TA probe (TA‐Nap) exhibited precise intracellular co‐localization with PLK1 (Figure 2E and Scheme S2). Cellular target engagement was further confirmed by Cellular Thermal Shift Assay (CETSA) and Drug Affinity Responsive Target Stability (DARTS) assays, both of which demonstrated that TA significantly enhanced the thermal stability and protease resistance of PLK1, respectively (Figure S2I,J). Quantitative binding affinities were determined using surface plasmon resonance (SPR) and microscale thermophoresis (MST). TA bound to full‐length PLK1 with K_d_ values of 7.59 µm (SPR) and 12.85 µm (MST) (Figure 2F and Figure S2K).

To identify the binding sites of TA on PLK1, we first performed photoaffinity labeling coupled with LC‐MS/MS analysis. Upon UV irradiation (365 nm), the diazirine‐containing TA probe (Scheme S3) covalently crosslinked to PLK1. Following mass spectrometric analysis, three labeled peptides were identified, corresponding to two potential binding pockets: pocket 1 (T551/T561) located at the junction between the kinase domain (CD) and polo‐box domain (PBD), and pocket 2 (T440/T513) within the PBD domain (Figure 2G,H). SPR and MST analysis confirmed that TA directly binds to both the kinase domain (PLK1‐CD, K_d_ = 33.69 µm) and the polo‐box domain (PLK1‐PBD, K_d_ = 9.03 µm) (Figure 2I and Figure S2K). These findings directed our attention to pocket 1 at the CD‐PBD interface. Based on the photoaffinity labeling sites, molecular docking simulations identified four spatially adjacent residues (R57, R135, W410, and F559) as candidate binding sites for TA (Figure 2J). To experimentally validate these predictions, we generated a series of point mutants in His‐MBP‐PLK1 and measured their binding affinities to TA by MST. Mutations of key residues in pocket 1 (R135A and W410A) dramatically reduced the binding affinity of TA (K_d_ increased from 3.98 ± 1.35 µm for wild‐type PLK1 to 295.80 ± 218.93 and 69.33 ± 29.95 µm, respectively), whereas mutation of F559A in the same pocket had minimal effect (Figure 2K). Collectively, these results indicate that TA binds to pocket 1 located at the kinase‐polo‐box domain junction of PLK1, with R135 and W410 serving as critical residues for the interaction.

### 2.3 TA Triggers PLK1‐Dependent Oxidative Stress, DNA Damage, and Mitochondrial Dysfunction to Induce Cell Death

To systematically elucidate how TA‐mediated PLK1 targeting leads to CRC cell death, we performed an integrated bioinformatics analysis (incorporating KEGG, GO‐CC, and Reactome databases) on the differentially expressed proteins identified by TMT‐based quantitative proteomics (TA vs. DMSO). Pathway enrichment analysis revealed a highly orchestrated, hierarchical cascade of cellular stress responses. As shown in Figure 3A, the perturbed pathways clustered into five logically interconnected functional modules. Mechanistically, TA induces cell cycle arrest by targeting PLK1. This initial blockade subsequently triggers severe oxidative stress, characterized by dysregulation of glutathione metabolism and reactive oxygen species (ROS) detoxification enzymes. Consequently, excessive intracellular ROS accumulation inflicts catastrophic secondary damage, evidenced by robust enrichment of DNA damage pathways (e.g., replication initiation inhibition and p53 signaling activation) and mitochondrial dysfunction (e.g., impaired oxidative phosphorylation and respiratory chain collapse). Ultimately, this unresolved genomic and organelle damage converges to trigger terminal cell death programs, including ferroptosis and apoptosis. To further dissect the specific molecular effectors driving this cascade, we mapped the regulatory network between significantly altered proteins and these five functional clusters using a chord diagram (Figure 3B). The expression landscape revealed robust bidirectional modulation: key regulators required for cell cycle progression and DNA replication (e.g., CDK1, KIF family proteins) were significantly downregulated (blue), whereas numerous effectors responding to severe oxidative stress and mediating cell death (e.g., FTL, HMOX1, CDKN1A) were substantially upregulated (red). Collectively, these global proteomic signatures delineate a sequential pathological cascade in which TA‐induced cell cycle arrest triggers ROS overproduction, ultimately leading to severe DNA and mitochondrial damage that drives CRC cell death.

**FIGURE 3:**
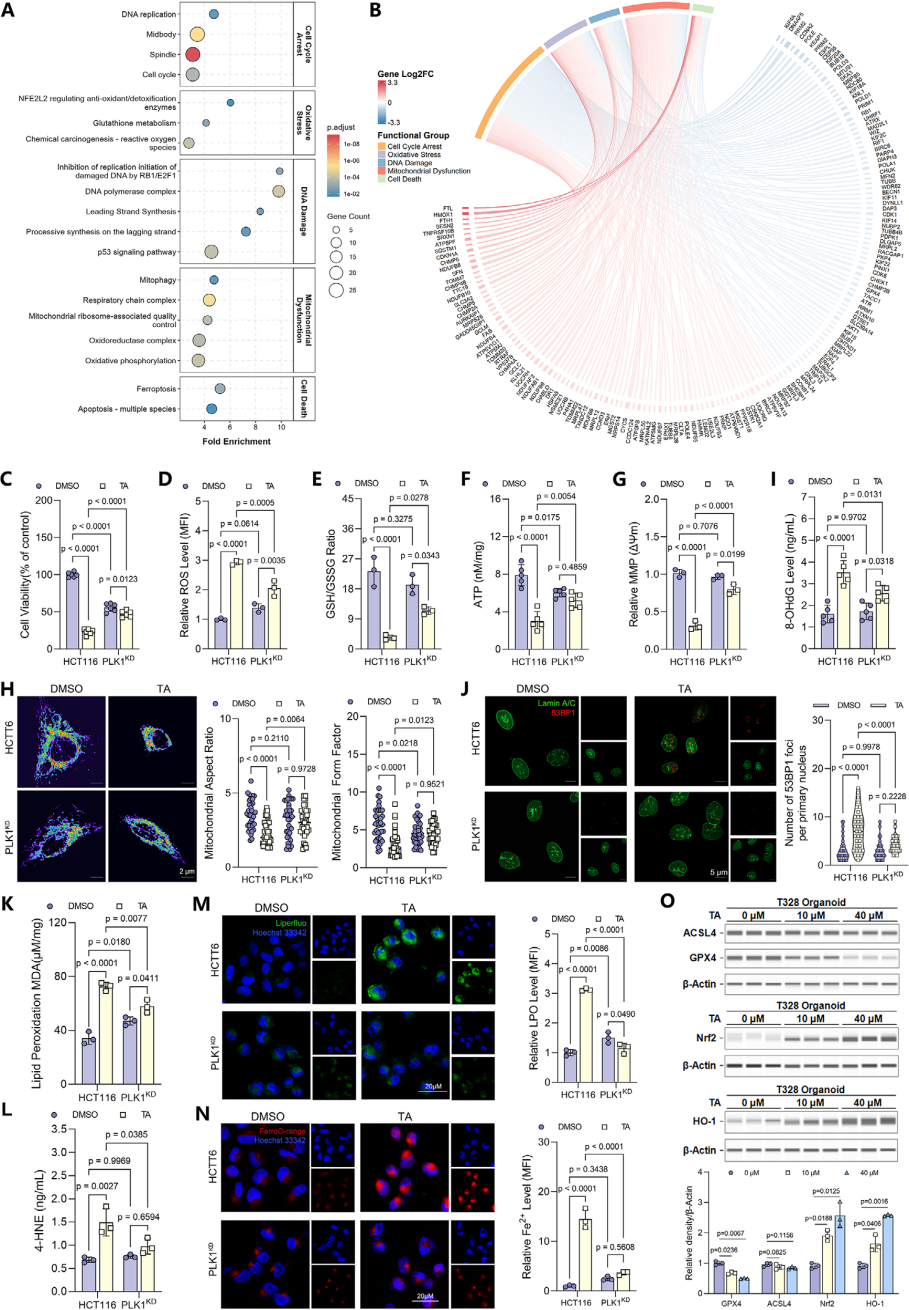
TA induces a PLK1‐dependent death‐inducing cascade involving oxidative stress, mitochondrial/DNA damage, and ferroptosis. (A) Bubble plot of enriched pathways from integrated KEGG, GO‐CC, and Reactome analysis in HCT116 cells (TA vs. DMSO). Bubble size corresponds to gene count, and color indicates adjusted p‐value. (B) Circos plot depicting the relationship between differentially expressed genes (Log_2_FC) and functional groups. Red and blue lines indicate up‐ and down‐regulated genes, respectively. (C) Cell viability of HCT116 and PLK1^KD^ cells treated with 1 µm TA or DMSO for 24 h (*n* = 6). (D) Relative ROS levels in HCT116 and PLK1^KD^ cells treated with 2 µm TA or DMSO for 12 h, quantified using DCFH‐DA probe and shown as mean fluorescence intensity (MFI) (*n* = 3). (E) The GSH/GSSG ratio was determined in HCT116 and PLK1^KD^ cells exposed to 2 µm TA or DMSO for 12 h (*n* = 3). (F) HCT116 and PLK1^KD^ cells were treated with 2 µm TA or DMSO for 12 h, and intracellular ATP levels were determined and normalized to protein concentration (nM/mg protein) (*n* = 5). (G) Measurement of mitochondrial membrane potential (ΔΨm) using the TMRM fluorescent probe in HCT116 and PLK1^KD^ cells treated with 2 µm TA or DMSO for 6 h (*n* = 3). (H) Representative confocal images of mitochondrial morphology (MitoTracker Deep Red) in HCT116 and PLK1^KD^ cells treated with DMSO or 1 µm TA for 6 h. Quantification of mitochondrial aspect ratio and mitochondrial form factor (*n* ≥ 37). Scale bar = 2 µm. (I) HCT116 and PLK1^KD^ were treated with 2 µm TA or DMSO for 12 h, and 8‐OHdG levels were measured in cell lysates by ELISA and expressed as ng/mL (*n* = 3). (J) Representative immunofluorescence images showing 53BP1 foci (red) and Lamin A/C (green) in HCT116 and PLK1^KD^ cells treated with DMSO or 1 µm TA for 6 h. Scale bar = 5 µm. Right panel shows the number of 53BP1 foci per primary nucleus (*n* ≥ 33). (K, L) Levels of MDA and 4‐HNE in HCT116 and PLK1^KD^ cells following treatment with 2 µm TA or DMSO for 24 h (*n* = 3). (M, N) Representative fluorescence images showing lipid ROS and intracellular ferrous iron levels in HCT116 and PLK1^KD^ cells treated with DMSO or 2 µm TA for 24 h. Lipid ROS was detected by Liperfluo (green), and Fe^2^^+^ was detected by FerroOrange (red). Nuclei were stained with Hoechst 33342 (blue). Scale bars = 20 µm. Lipid ROS and intracellular Fe^2^^+^ levels were quantified based on MFI (*n* = 3). (C–N) Statistical significance was determined by two‐way ANOVA followed by Šídák's multiple comparisons test. (O) Automated capillary Western blot analysis of ferroptosis‐related proteins in T328 patient‐derived CRC organoids treated with 0, 10, and 40 µm TA for 24 h. Protein expression was measured by chemiluminescence and quantified as the area under the curve. The corresponding signal peak data and quantified areas are provided in Figure S4 and Table S2. Data are presented as mean ± s.d. Statistical analysis was performed using one‐way ANOVA followed by Tukey's multiple comparisons test. Exact *p*‐values are indicated in the figures.

Guided by these findings, we systematically validated the downstream death‐inducing cascade. To confirm that TA‐mediated perturbation of these biological processes depends on PLK1, we generated a stable PLK1‐knockdown CRC cell line (HCT116 PLK1^KD^). Functionally, TA significantly suppressed cell viability and clonogenicity, whereas PLK1 knockdown restored cell survival (Figure 3C and Figure S3A,B), supporting a PLK1‐dependent cytotoxic mechanism. Phenotypically, TA provoked severe oxidative stress, characterized by excessive ROS accumulation and marked GSH depletion (Figure 3D,E). Excessive ROS further triggered severe mitochondrial structural and functional impairment, as evidenced by pronounced mitochondrial fragmentation, loss of mitochondrial membrane potential, and ATP depletion (Figure 3F–H). Concurrently, excessive ROS accumulation induced genomic instability, elevated oxidative DNA damage (8‐OHdG), and extensive 53BP1 foci formation (Figure 3I,J). The TA‐induced effects on the above biological processes were significantly attenuated in HCT116 PLK1^KD^ cells, confirming PLK1 as a critical upstream regulator of this signaling cascade.

Accumulating evidence indicates that unresolved oxidative stress, together with catastrophic mitochondrial dysfunction, can exhaust cellular antioxidant defense systems (such as the GSH pool), thereby driving cells toward iron‐dependent lipid peroxidation and ferroptosis [29, 30]. We found that TA treatment induced significant lipid peroxidation, reflected by elevated malondialdehyde (MDA), 4‐hydroxynonenal (4‐HNE), and Liperfluo signals, along with intracellular iron overload (Figure 3K–N). At the molecular level, TA dose‐dependently downregulated the anti‐ferroptotic core enzyme GPX4 while upregulating Nrf2 and HO‐1 in PDOs and in vivo xenograft tissues (Figure 3O and Figure S3C,D). Crucially, this ferroptotic signature was effectively reversed by PLK1 knockdown (Figure S3E). Co‐treatment with the pharmacological ferroptosis inhibitor Ferrostatin‐1 (Fer‐1) robustly mitigated TA‐induced iron accumulation, lipid peroxidation, and dysregulation of marker proteins (Figure S3F–H). Collectively, these data demonstrate that TA initiates a PLK1‐dependent cascade culminating in mitochondrial dysfunction, DNA damage, and ferroptosis.

### 2.4 PLK1 is Associated with L‐OHP Resistance in CRC

Upon evaluating the PLK1 expression profiles in our established PDOs, we identified a striking negative correlation between PLK1 transcript levels and L‐OHP sensitivity (R = −0.53) (Figure 4A). This observation was further corroborated in a large panel of colorectal adenocarcinoma (COREAD) cell lines, where higher PLK1 expression was significantly associated with reduced L‐OHP potency (Figure 4B). Kaplan‐Meier survival analysis of the TCGA‐CRC cohort demonstrated that high PLK1 expression is significantly associated with unfavorable overall survival (Figure 4C). Furthermore, CRISPR‐Cas9 dependency mapping (DepMap/Chronos) identified PLK1 as a pan‐essential gene for cell survival across diverse COREAD lineages (Figure 4D). Immunohistochemical (IHC) staining confirmed that PLK1 protein expression was upregulated in L‐OHP‐resistant HCT116‐OR cells relative to parental HCT116 cells (Figure 4E and Figure S5A). Crucially, ectopic overexpression of PLK1 in parental HCT116 cells sufficiently conferred resistance to L‐OHP, phenocopying the OR trait. Conversely, targeted knockdown of PLK1 in HCT116‐OR cells drastically resensitized these refractory cells to L‐OHP‐induced cytotoxicity (Figure 4F). Collectively, these findings demonstrate that elevated PLK1 expression is strongly associated with L‐OHP resistance in CRC and plays a critical role in maintaining the resistant phenotype.

**FIGURE 4:**
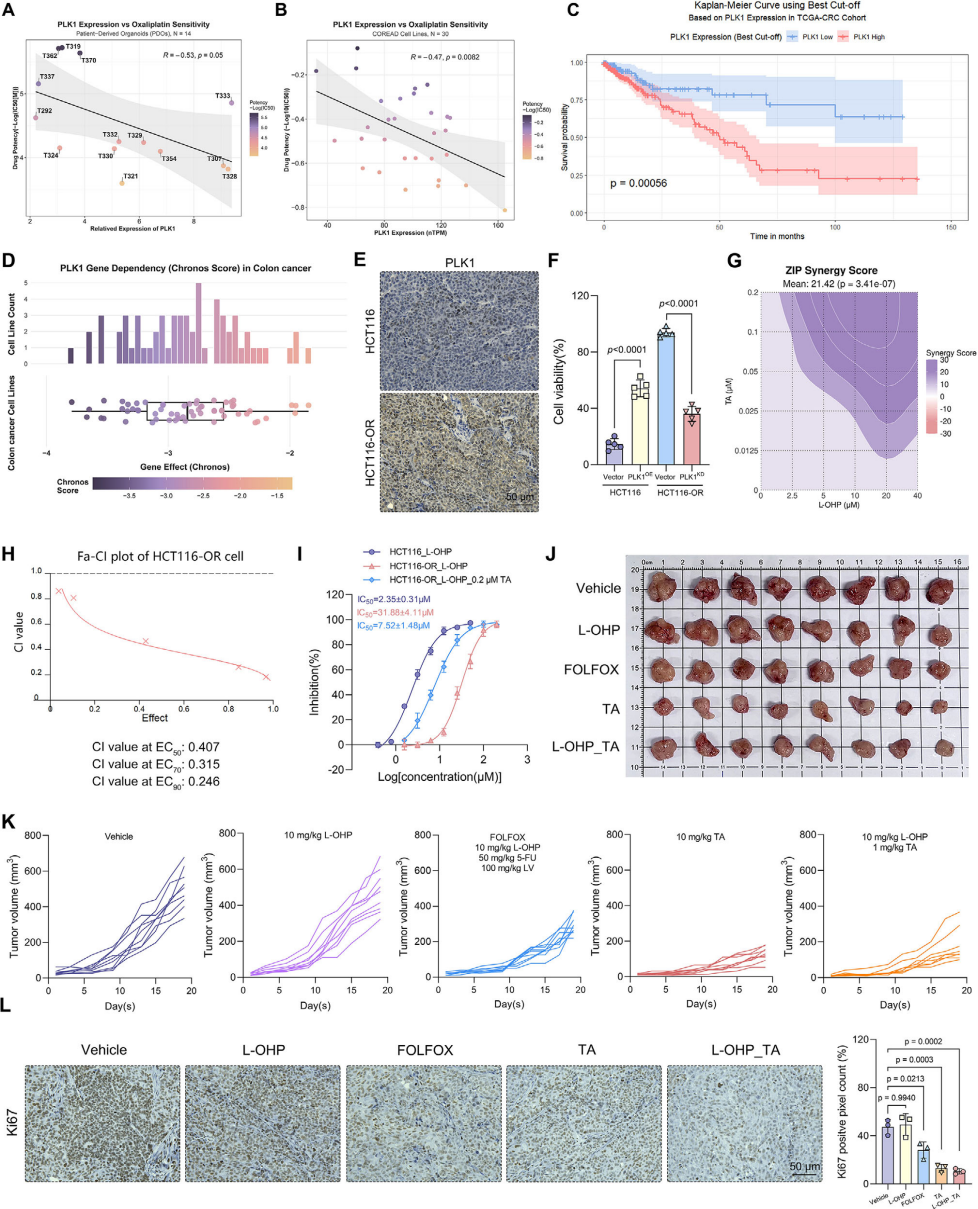
PLK1 drives L‐OHP resistance in CRC, whereas TA synergizes with L‐OHP to overcome resistance. (A) Pearson correlation analysis between PLK1 expression and L‐OHP sensitivity in PDOs (*n* = 14). PLK1 relative expression was plotted against L‐OHP potency (‐Log_10_(IC_50_)). The correlation was determined by Pearson's correlation coefficient (R = −0.53). (B) Pearson correlation analysis between PLK1 mRNA expression and L‐OHP sensitivity in 30 COREAD cell lines. PLK1 expression (nTPM) from the Human Protein Atlas (HPA) database was plotted against L‐OHP potency (−Log_10_(IC_50_)) from the Genomics of Drug Sensitivity in Cancer (GDSC) database. A significant negative Pearson correlation was observed (R = −0.47). (C) Kaplan‐Meier curves of overall survival in the TCGA‐CRC cohort based on PLK1 expression (log‐rank test). (D) CRISPR‐Cas9 dependency mapping (DepMap) illustrating the Chronos score of PLK1 across diverse CRC cell lines. The upper panel displays the frequency distribution of PLK1 Chronos scores across CRC cell lines, and the lower panel shows a box‐and‐whisker plot overlaid with individual cell line data points. The color gradient reflects the intensity of the Chronos score. (E) IHC staining showed the expression level of PLK1 in HCT116‐derived and HCT116‐OR‐derived xenograft tumor tissues. Scale bar, 50 µm. (F) Effect of PLK1 overexpression and knockdown on cell viability (*n* = 5). (G) The synergistic interaction between TA and L‐OHP was evaluated using the ZIP model in SynergyFinder. Synergy scores above 10 are generally considered synergistic. (H) The combination effect of TA and L‐OHP was evaluated using the Chou‐Talalay method. The Fa‐CI plot illustrates the CI values across a range of fractional effects. (I) HCT116 cells and HCT116‐OR cells co‐treated with 0.2 µm TA were exposed to increasing concentrations of L‐OHP for 24 h, after which cell viability was assessed (*n* = 3). (J) Representative photographs of the excised xenograft tumors from mice treated with Vehicle, 10 mg/kg L‐OHP (twice a week), FOLFOX (10 mg/kg L‐OHP, 50 mg/kg 5‐FU and 100 mg/kg Leucovorin Calcium (LV), all twice a week), 10 mg/kg TA (once every two days), or L‐OHP_TA (10 mg/kg L‐OHP twice a week and 1 mg/kg TA once every two days) (*n* = 9). (K) Individual tumor growth trajectories per group. (L) IHC staining and quantification of Ki67. Scale bar, 50 µm. Data are presented as mean ± s.d. Statistical analysis was performed using one‐way ANOVA followed by Tukey's multiple comparisons test. Exact p‐values are indicated in the figures.

### 2.5 TA Synergizes with L‐OHP to Overcome Chemoresistance in CRC

Previous studies have shown that PLK1 inhibitors have demonstrated potential to reverse platinum resistance in both clinical and preclinical settings, not only exerting synergistic anticancer effects with L‐OHP but also improving progression‐free survival and objective response rates in patients with platinum‐refractory ovarian cancer and head and neck squamous cell carcinoma [31, 32, 33, 34]. We subsequently evaluated the anti‐proliferative activity of TA in HCT116‐OR cells. TA exhibited potent cytotoxicity with an IC_50_ value of 1.251 µm (Figure S5B), and showed significant synergistic effects with L‐OHP at doses with no obvious toxicity (Figure 4G). Combination index (CI) analysis further confirmed significant synergism, with CI values of 0.407, 0.315, and 0.246 at EC_50_, EC_70_, and EC_90_, respectively (Figure 4H and Figure S5C). Co‐treatment with 0.2 µm TA significantly shifted the dose‐response curve of L‐OHP to the left and reduced the IC_50_ from 31.88 ± 4.11 to 7.52 ± 1.48 µm, yielding a reversal index (RI) of 4.24 (Figure 4I). Consistently, TA significantly resensitized HCT‐8‐OR cells to L‐OHP, reducing its IC_50_ from 29.14 ± 9.17 to 5.33 ± 2.69 µm (RI = 5.47) (Figure S5D). PLK1 knockdown partially attenuated the cytotoxic effect of TA/L‐OHP co‐treatment, whereas PLK1 re‐expression restored sensitivity to the combination in both HCT116‐OR and HCT‐8‐OR cells, providing additional support for the involvement of PLK1 in TA‐mediated chemosensitization (Figure S5E,F). In addition, TA showed relatively limited effects on the viability of normal human colonic epithelial NCM460 cells at concentrations up to 12.5 µm (Figure S5G), compared with the 0.2 µm concentration used for chemosensitization. In vivo, both high‐dose TA (10 mg/kg) monotherapy and the combination of low‐dose TA (1 mg/kg) with L‐OHP (10 mg/kg) exhibited substantial antitumor effects in HCT116‐OR xenograft models without causing significant body weight loss. Under the treatment conditions used in this HCT116‐OR xenograft model, the TA + L‐OHP combination resulted in greater tumor growth inhibition than the FOLFOX comparator group (Figure 4J, K and Figure S5H–J). IHC analysis of tumor tissues revealed that high‐dose TA and the L‐OHP + TA combination significantly reduced Ki67 proliferation index compared with other groups (Figure 4L). Collectively, these findings highlight the synergistic interaction between TA and L‐OHP and suggest the potential of PLK1 inhibition as a strategy for resensitizing L‐OHP‐resistant CRC to chemotherapy.

### 2.6 PLK1 Regulates Vesicle‐Associated Damage Handling in L‐OHP‐Resistant CRC

To elucidate the clinical relevance and underlying molecular mechanisms of PLK1‐driven L‐OHP resistance, we integrated seven independent CRC transcriptomic cohorts (e.g., GSE81653) (Figure S6), encompassing a total of 408 patient samples [35, 36, 37, 38, 39, 40, 41, 42]. Patients were stratified into Responder and Non‐Responder groups based on RECIST criteria and progression‐free survival. PLK1 expression was significantly elevated in the Non‐Responder group (Figure 5A) and exhibited moderate predictive value for chemoresistance (AUC = 0.701) (Figure 5B). By comparison, the representative proliferation markers MKI67 and PCNA showed limited discriminatory ability in the same cohort (AUC = 0.520 and 0.519, respectively; Figure S7A), suggesting that the association between PLK1 and chemoresistance may not be solely attributable to general proliferative activity. Given the profound intrinsic heterogeneity of clinical chemoresistance, the initial Orthogonal Partial Least Squares Discriminant Analysis (OPLS‐DA) utilizing the entire cohort failed to yield distinct group separation (Figure 5C and Figure S7B). To enhance the signal‐to‐noise ratio and pinpoint PLK1‐associated resistance signatures, we employed an extreme phenotype enrichment strategy. By isolating a core cohort (*n* = 245) comprising the 60% of Non‐Responders with the highest PLK1 expression and the 60% of Responders with the lowest PLK1 expression, the refined OPLS‐DA revealed distinct transcriptomic profiles between sensitive and resistant phenotypes (Figure 5D and Figure S7C). These findings further support the clinical relevance of PLK1 as a key molecule associated with chemoresistance and a potential biomarker in CRC.

**FIGURE 5:**
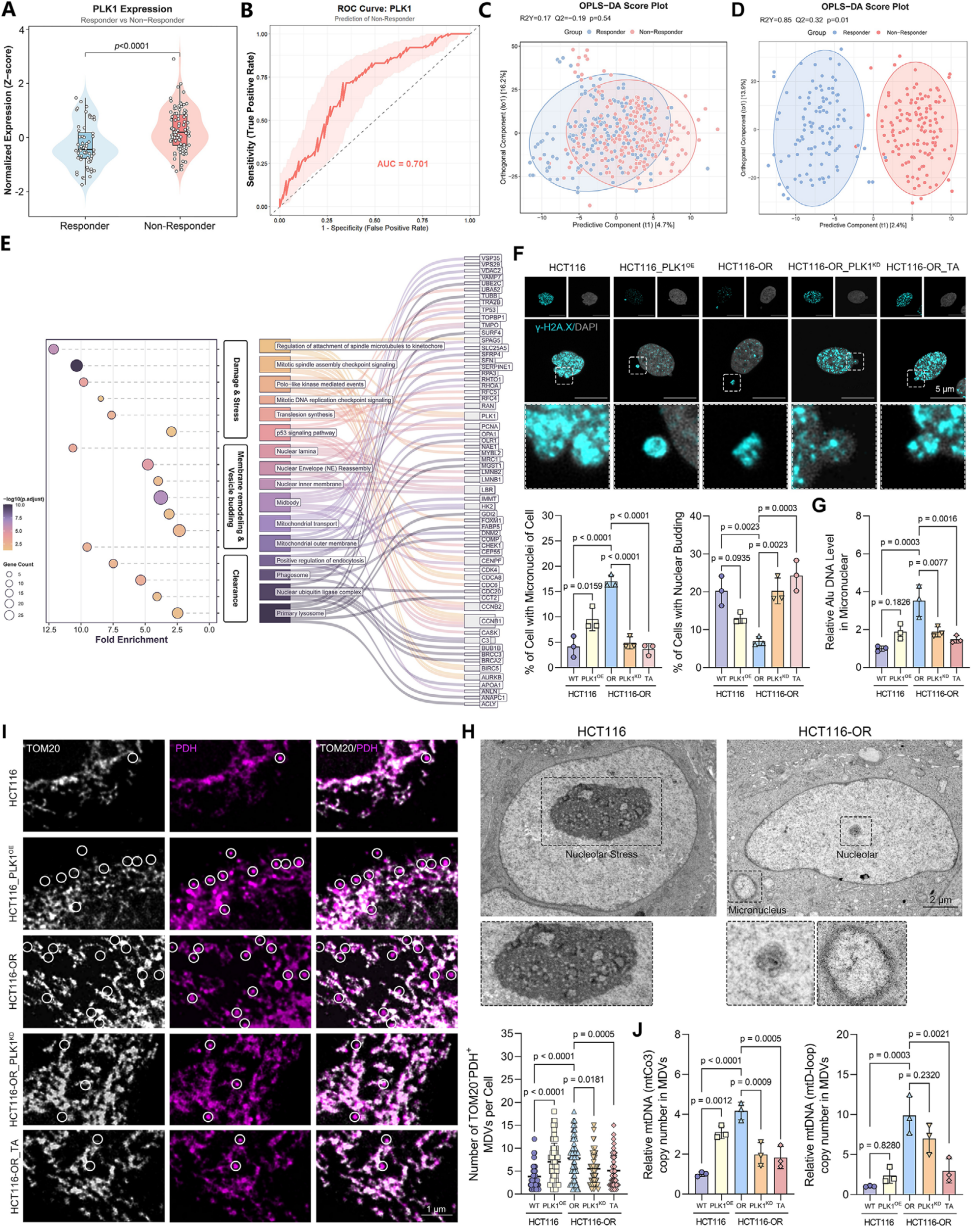
PLK1 contributes to L‐OHP resistance in CRC through vesicle‐associated damage clearance. (A) Violin plot showing relative PLK1 expression levels in CRC patients stratified into Responder and Non‐Responder groups across integrated clinical cohorts (*n* = 408). (B) Receiver Operating Characteristic (ROC) curve evaluating the predictive efficacy of PLK1 expression for chemoresistance (AUC = 0.701). (C, D) OPLS‐DA score plots of the entire clinical cohort (C, *n* = 408) and the refined core cohort (D, *n* = 245) with TOP 2000 MAD. (E) Sankey/Bubble plot illustrating the integrative pathway enrichment analysis (KEGG, GO, Reactome) of the differentially expressed genes from the core cohort. (F, G, I, J) HCT116, PLK1^OE^ HCT116, HCT116‐OR, and PLK1^KD^ HCT116‐OR cells were treated with 10 µm L‐OHP for 3 h, with 0.5 µm TA co‐treatment as indicated. (F) Representative immunofluorescence images and quantitative bar charts (lower panels) (*n* = 3). Scale bar, 50 µm. (G) qPCR quantification of nuclear‐specific Alu DNA sequences in isolated micronuclear fractions across indicated groups after treatment with L‐OHP for 6 h (*n* = 3). (H) TEM images of nuclear morphology in HCT116 and HCT116‐OR tumor xenografts following treatment with L‐OHP. Scale bar, 2 µm. (I) Immunofluorescence of TOM20^−^/PDH^+^ MDVs. Quantification of TOM20^−^/PDH^+^ MDVs per cell is shown (*n* ≥ 30). Scale bar, 1 µm. (J) Relative *mtCo3* and *mtD‐loop* copy numbers quantified by qPCR in subcellular vesicular fractions isolated by ultracentrifugation and verified after treatment with L‐OHP for 6 h (*n* = 3). Data are presented as mean ± s.d. Statistical analysis was performed using one‐way ANOVA followed by Tukey's multiple comparisons test. Exact *p*‐values are indicated in the figures.

Subsequent integrative pathway enrichment analysis (KEGG, Reactome, and GO‐CC) mapped these transcriptomic alterations to a sequential, three interconnected functional modules: “Damage & Stress”, “Membrane remodeling & Vesicle budding”, and “Clearance” (Figure 5E and Figure S7D). This suggests that PLK1 may act as a key modulator associated with vesicle extrusion and clearance processes to maintain cellular homeostasis under chemotherapy stress. Subsequently, we systematically validated this hypothesis. Immunofluorescence revealed that, following L‐OHP treatment, both HCT116‐OR and PLK1^OE^ HCT116 cells exhibited increased nuclear budding associated with γ‐H2A.X^+^ damaged chromatin‐containing micronuclei. In contrast, PLK1 knockdown or TA treatment reduced the occurrence of these budding structures and was accompanied by increased retention of damaged chromatin within the nucleus (Figure 5F and Figure S7E). To further assess the redistribution of nuclear DNA into micronuclear fractions, we isolated subcellular fractions and quantified nuclear‐derived Alu DNA sequences. TA treatment or PLK1 knockdown significantly reduced Alu DNA abundance in the micronuclear fraction (Figure 5G), consistent with reduced nuclear damage extrusion. A similar phenotype was observed in L‐OHP‐resistant HCT‐8 cells, which exhibited increased micronuclear DNA levels that were significantly reduced by TA treatment (Figure S7F). In L‐OHP‐treated resistant HCT116‐OR cells, transmission electron microscopy (TEM) further revealed distinct cytoplasmic micronuclear structures in L‐OHP‐treated HCT116‐OR cells (Figure 5H).

In addition to inducing DNA crosslinking that leads to replication fork stalling and severe double‐strand breaks, L‐OHP disrupts ribosome biogenesis, triggering pronounced nucleolar stress [43, 44, 45]. Consistently, HCT116 cells displayed prominent irregular and electron‐dense nucleolar structures, whereas HCT116‐OR cells maintained relatively compact nucleoli within the main nucleus (Figure 5H). Together, these observations suggest that nuclear budding and micronuclear formation may contribute to the segregation and removal of damaged chromatin in resistant cells.

A similar PLK1‐associated process was observed for mitochondrial damage. HCT116‐OR and PLK1^OE^ HCT116 cells exhibited increased formation of TOM20^−^/PDH^+^ MDVs (Figure 5I and Figure S7G), which was reduced by TA treatment or PLK1 knockdown. Furthermore, qPCR analysis of isolated vesicular fractions showed increased enrichment of *mtCo3*, *mtND1*, and *mtD‐loop* DNA in resistant cells, which was reduced following PLK1 knockdown or TA treatment (Figure 5J and Figure S7H). Similar increases in TOM20^−^/PDH^+^ MDV formation and vesicular mtDNA abundance were observed in HCT‐8‐OR cells and were attenuated by TA (Figure S7I,J).

Taken together, these findings support a model in which PLK1‐associated nuclear budding and MDV formation contribute to vesicular damage handling in L‐OHP‐resistant cells, while PLK1 knockdown or TA treatment impairs these processes and promotes intracellular damage accumulation.

### 2.7 PLK1‐Mediated CEP55 Ser436 Phosphorylation Facilitates ESCRT Recruitment During Vesicle‐Associated Damage Clearance

Having observed that nuclear budding and MDV release are associated with damage handling in resistant cells, we next investigated how elevated PLK1 activity regulates these processes. A significant enrichment of the ESCRT pathway was observed following TA treatment (Figure S8A). The ESCRT machinery is classically responsible for catalyzing topologically complex membrane scission events, and recent studies have expanded its role to include nuclear envelope remodeling and micronuclei formation [46, 47]. Consequently, we hypothesized that PLK1 may facilitate vesicular damage clearance through regulation of ESCRT‐mediated membrane scission. Immunofluorescence imaging demonstrated that CHMP4B, a core ESCRT‐III subunit, localized to the neck of nuclear buds (Figure 6A) and was enriched on mitochondria (Figure 6B and Figure S8B) in HCT116‐OR cells. Crucially, both pharmacological inhibition of PLK1 by TA and genetic knockdown significantly suppressed CHMP4B recruitment to these vesicular structures, supporting a role for PLK1 in ESCRT recruitment. Similar CHMP4B recruitment to nuclear budding sites and mitochondria was observed in HCT‐8‐OR cells, both of which were attenuated by TA treatment (Figure S8C,D).

**FIGURE 6:**
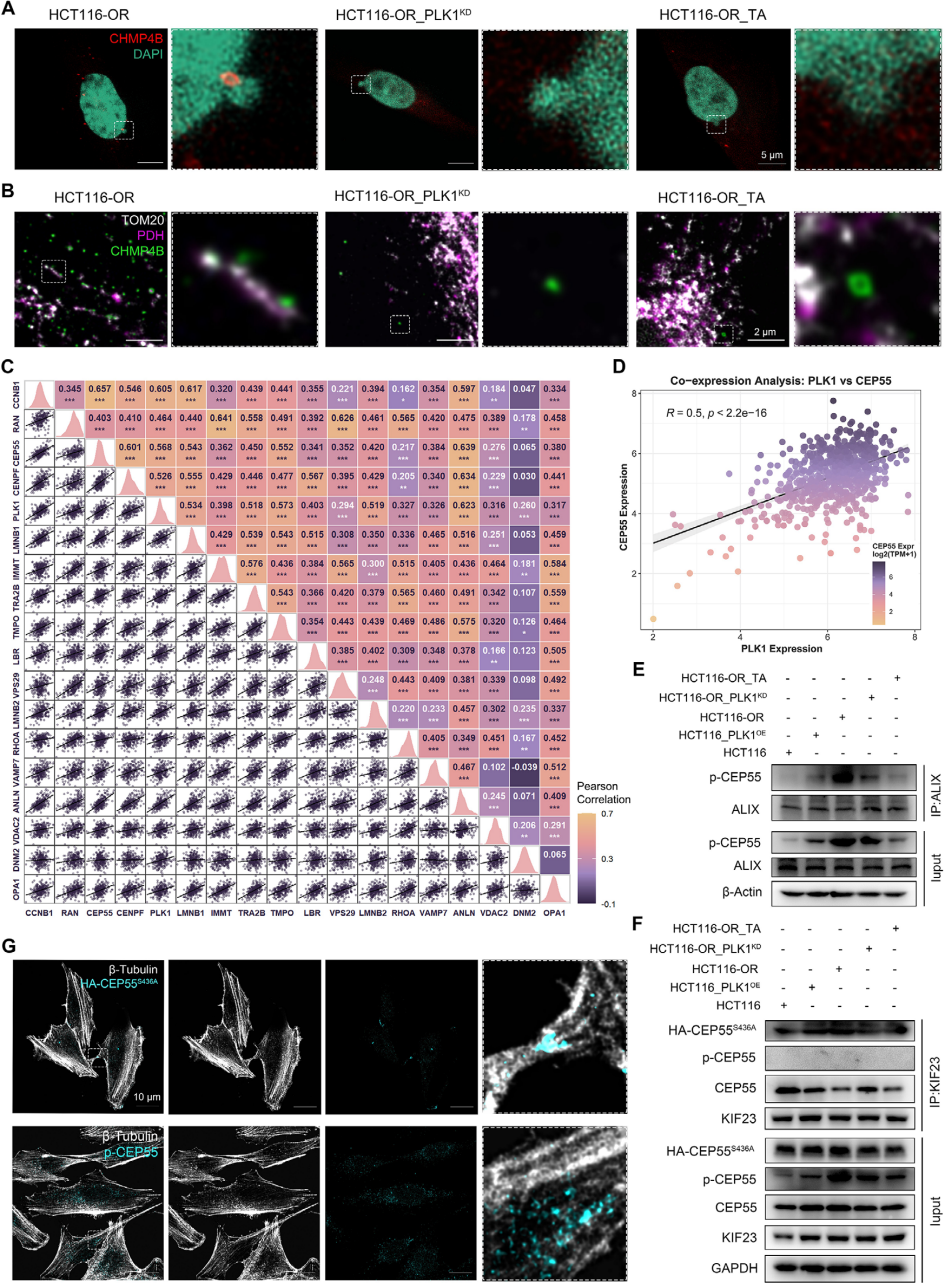
TA disrupts the PLK1–CEP55–ESCRT scission machinery to prevent damage clearance. (A, B) Immunofluorescence images showing the enrichment of the ESCRT‐III subunit CHMP4B at the nuclear budding neck (A) and mitochondria (B) in HCT116‐OR and PLK1^KD^ HCT116‐OR cells treated with 10 µm L‐OHP for 3 h, with 0.5 µm TA co‐treatment as indicated. Scale bar, 5 or 2 µm, respectively. (C) The matrix integrates pairwise scatter plots with linear regression lines (lower‐left triangle), gene expression density distributions (diagonal), and a heatmap of Pearson correlation coefficients (R) (upper‐right triangle). The color gradient corresponds to the strength and direction of the correlation. Statistical significance is denoted by asterisks (^*^ *p* < 0.05, ^**^ *p* < 0.01, ^***^ *p* < 0.001). (D) Scatter plot showing a moderate positive correlation between PLK1 and CEP55 transcript levels across cancer cell lines from the DepMap database (R = 0.5). Exact p‐values are indicated in the figures. (E, F) HCT116, PLK1^OE^ HCT116, HCT116‐OR, PLK1^KD^ HCT116‐OR, and TA‐treated HCT116‐OR cells were treated with 10 µm L‐OHP for 12 h, with 0.5 µm TA co‐treatment as indicated. (E) Co‐IP analysis of the association between ALIX and p‐CEP55. (F) Co‐IP analysis of the association of KIF23 with CEP55, p‐CEP55, or CEP55^S436A^. (G) Immunofluorescence images illustrating the distinct subcellular localizations of the HA‐CEP55^S436A^ and p‐CEP55 (cyan). Scale bar, 10 µm.

To delineate the molecular link between PLK1 and the ESCRT machinery, we analyzed differentially expressed genes associated with the “Damage & Stress” and “Membrane remodeling & Vesicle budding” modules in our clinical resistance cohort. We identified a positive correlation between the expression of PLK1 and CEP55 (Figure 6C and Figure S8E), a known ESCRT‐interacting adaptor. To assess whether this association extends beyond our clinical cohort, we performed large‐scale co‐expression analyses utilizing the broad cancer cell line panel from the DepMap database. A significant positive correlation between PLK1 and CEP55 was also identified (Figure 6D). During canonical cytokinesis, CEP55 recruits the ESCRT‐I component TSG101 and the accessory protein ALIX via its hinge region to execute midbody abscission [48]. Previous studies establish that PLK1‐mediated phosphorylation of CEP55 at Ser436 induces a conformational shift that prevents its premature binding to the centralspindlin component MKLP1 (KIF23) [49]. Western blot analysis confirmed that the PLK1‐mediated phosphorylation of CEP55 at Ser436 was substantially elevated in HCT116‐OR and PLK1^OE^ HCT116 cells, but was potently suppressed by TA treatment or PLK1 knockdown (Figure S8F,G). A similar increase in CEP55 Ser436 phosphorylation was observed in HCT‐8‐OR cells and was attenuated by TA treatment (Figure S8H). To determine whether TA directly inhibits PLK1 catalytic activity, we performed an in vitro kinase assay using a CEP55‐derived peptide containing Ser436. Recombinant PLK1 efficiently phosphorylated CEP55‐WT, whereas the S436A mutation significantly diminished kinase activity, supporting Ser436‐dependent phosphorylation (Figure S8I). TA dose‐dependently inhibited PLK1 activity, with comparable IC_50_ values at 50 and 200 µm ATP (2.239 ± 0.449 and 2.337 ± 0.482 µm, respectively) (Figure S8J), suggesting a non‐ATP‐competitive mechanism consistent with its binding to the kinase domain–polo‐box domain interface rather than the canonical ATP‐binding pocket.

However, whether this specific phosphorylation event impacts CEP55's interaction with the ESCRT‐associated proteins, ALIX and TSG101, has remained unclear. Given that CEP55 specifically recruits ALIX and TSG101 via its N‐terminal hinge region (amino acid residues 160‐217), which is structurally and spatially distinct from the C‐terminal Ser436 phosphorylation site, we reasoned that Ser436 phosphorylation may regulate the availability or subcellular distribution of CEP55 for ESCRT recruitment rather than directly disrupting its ESCRT‐binding interface. Co‐IP experiments further supported this possibility. The association of p‐CEP55 with ALIX was enhanced in L‐OHP‐resistant and PLK1^OE^ cells but attenuated by TA treatment (Figure 6E). Consistently, an enhanced association between p‐CEP55 and TSG101 was observed in HCT116‐OR cells and was similarly reduced by TA treatment (Figure S8K). Conversely, when PLK1 was inhibited by TA or genetically silenced, CEP55 exhibited enhanced association with KIF23, consistent with that observed for the phospho‐deficient HA‐CEP55^S436A^ mutant (Figure 6F). Immunofluorescence imaging revealed that the HA‐CEP55^S436A^ was prominently enriched at the midbody, structurally marked by the β‐tubulin‐containing intercellular bridge, while p‐CEP55 displayed a broadly distributed punctate pattern throughout the cytoplasm (Figure 6G).

To further examine the functional role of CEP55 Ser436 phosphorylation in damage extrusion, we performed rescue experiments using CEP55‐knockdown (CEP55^KD^) HCT116‐OR cells reconstituted with HA‐CEP55, the phospho‐deficient HA‐CEP55^S436A^ mutant, or the phosphomimetic HA‐CEP55^S436D^ mutant. CEP55 knockdown reduced micronuclear DNA levels, TOM20^−^/PDH^+^ MDV formation, and vesicular mtDNA abundance, all of which were substantially restored by CEP55 re‐expression (Figure S9A–C). In contrast, CEP55^S436A^ failed to efficiently restore these phenotypes, whereas CEP55^S436D^ partially rescued nuclear and mitochondrial damage extrusion (Figure S9A–C). Consistently, CEP55 and CEP55^S436D^ restored CHMP4B recruitment to mitochondria and nuclear budding sites more effectively than CEP55^S436A^ (Figure S9D,E). These rescue experiments provide functional evidence that CEP55 S436 phosphorylation contributes to efficient ESCRT recruitment and vesicle‐mediated damage extrusion.

Together, these findings support a model in which elevated PLK1 activity in L‐OHP‐resistant cells promotes CEP55 Ser436 phosphorylation, limiting its association with KIF23 and thereby favoring its availability for ESCRT recruitment at nuclear and mitochondrial budding sites. TA disrupts this process by inhibiting PLK1 kinase activity, reducing CEP55 Ser436 phosphorylation and thereby attenuating ESCRT‐dependent vesicle‐mediated damage clearance.

### 2.8 TA Inhibits the PLK1‐CEP55‐ESCRT Axis Leading to Nuclear Damage Accumulation and Mitochondrial Dysfunction

To further examine whether disruption of PLK1‐mediated CEP55 phosphorylation contributes to impaired damage clearance, we performed rescue experiments using CEP55^KD^ HCT116‐OR cells reconstituted with either HA‐CEP55 or HA‐CEP55^S436A^. High‐resolution imaging of the nuclear envelope (Lamin A/C) revealed detached micronuclear structures in resistant cells, whereas TA‐treated or PLK1^KD^ HCT116‐OR cells, as well as CEP55^S436A^‐expressing cells, showed an increased frequency of nuclear buds that remained connected to the primary nucleus (Figure S10A). This phenotype was accompanied by increased retention of damaged chromatin within the nucleus, as indicated by increased 53BP1 foci (Figure 7A), together with nucleolar stress characterized by UBF1‐positive nuclear caps (Figure 7B). In parallel, impaired damage clearance was associated with mitochondrial dysfunction. TA‐treated or PLK1^KD^ HCT116‐OR cells, as well as CEP55^S436A^‐rescued cells, exhibited increased mitochondrial fragmentation, as reflected by reduced mitochondrial form factor and aspect ratio (Figure 7C and Figure S10B), together with increased mtROS (Figure 7D), reduced mitochondrial membrane potential (Figure 7E), and decreased intracellular ATP levels (Figure 7F). The similar phenotypes observed following TA treatment, PLK1 knockdown, and expression of CEP55^S436A^ further support the functional involvement of the PLK1–CEP55^S436^ axis in these processes.

**FIGURE 7:**
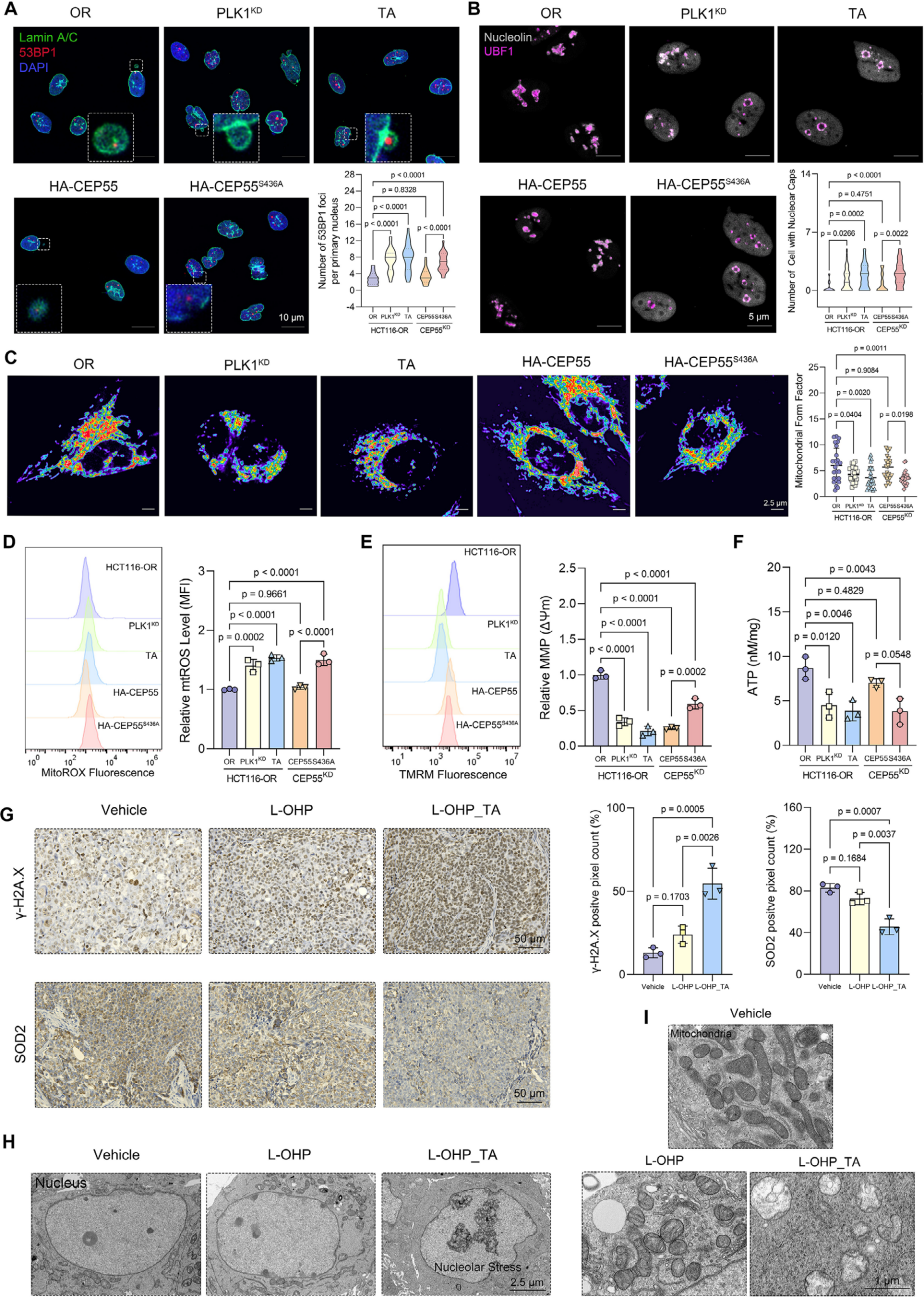
Blockade of PLK1‐CEP55‐mediated clearance induces genomic instability and mitochondrial dysfunction. (A‐C) Representative confocal immunofluorescence images of HCT116‐OR, PLK1^KD^ HCT116‐OR, and CEP55^KD^ HCT116‐OR cells rescued with HA‐CEP55 or HA‐CEP55^S436A^, following treatment with 10 µm L‐OHP for 3 h, with 0.5 µm TA co‐treatment as indicated. (A) Staining for Lamin A/C (green), 53BP1 (red), and DAPI (blue). Scale bars, 5 µm. Quantification of the number of 53BP1 foci per primary nucleus is shown on the right (*n* ≥ 25). (B) Staining for Nucleolin (gray) and UBF1 (magenta). Scale bar, 5 µm. Quantification of the number of cells with nucleolar caps is shown on the right (*n* ≥ 33). (C) Mitochondrial morphology visualized by immunofluorescence with color‐coded form factor analysis (blue to red indicating increasing mitochondrial elongation and network complexity). Scale bars, 2.5 µm. Quantification of mitochondrial form factor is shown on the right (*n* ≥ 25). (D–F) Flow cytometric analysis and biochemical assays of mitochondrial function in HCT116‐OR, PLK1^KD^ HCT116‐OR, and CEP55^KD^ HCT116‐OR cells rescued with HA‐CEP55 or HA‐CEP55^S436A^, following treatment with 10 µm L‐OHP for 12 h, with 0.5 µm TA co‐treatment as indicated. Representative flow cytometric histograms (left) and quantification (right) of mitochondrial ROS levels (D) and mitochondrial membrane potential (E) (*n* = 3). (F) Quantification of cellular ATP levels (nM/mg protein) (*n* = 3). (G) IHC staining and quantification of γ‐H2A.X and SOD2 in tumor sections from HCT116‐OR xenografts after the indicated treatments (*n* = 3). Scale bar, 50 µm. (H, I) TEM images showing ultrastructural changes in HCT116‐OR tumor xenografts after the indicated treatments. (H) Nuclear morphology. Scale bar, 2.5 µm. (I) Mitochondrial morphology. Scale bar, 1 µm. Data in (A‐G) are presented as mean ± s.d. Statistical significance was assessed by one‐way ANOVA followed by Tukey's multiple comparisons test. Exact p‐values are indicated in the figures.

Because autophagy may operate in parallel with MDV‐mediated damage processing, we next examined autophagic flux during the early treatment window. Analysis of LC3B and p62 in the presence of lysosomal inhibition after treatment with DMSO, 0.5 µm TA, or 0.5 µm TA combined with 10 µm L‐OHP for 3 h revealed no substantial alteration in autophagic flux under these conditions (Figure S10C). These results suggest that autophagy is not prominently activated during this early treatment window, although its potential contribution to damage processing cannot be excluded.

We further distinguished the direct effects of low‐dose TA from the downstream cellular consequences associated with impaired clearance of L‐OHP‐induced damage. After 24 h, 0.2 µm TA alone did not appreciably alter lipid peroxidation, intracellular Fe^2^^+^ levels, cell‐cycle distribution, or apoptosis. In contrast, co‐treatment with 0.2 µm TA and 10 µm L‐OHP resulted in G2/M accumulation and increased apoptosis (Figure S10D–H). These findings suggest that low‐dose TA does not itself induce detectable mitotic arrest or overt cell death under these conditions, but may enhance the response of resistant cells to L‐OHP‐induced damage by impairing damage clearance.

Consistent with the in vitro blockage of damage clearance, IHC staining of tumor tissues revealed that the combination of L‐OHP and TA induced accumulation of γ‐H2A.X and reduction of SOD2 (Figure 7G). TEM analysis further revealed altered nucleolar morphology, including nucleolar fragmentation and electron‐dense aggregates (Figure 7H), as well as swollen mitochondria with disrupted cristae architecture (Figure 7I) in tumors treated with high‐dose TA or the combination of TA and L‐OHP. Collectively, our in vitro and in vivo findings indicate that TA attenuates L‐OHP resistance by interfering with PLK1–CEP55–ESCRT‐associated vesicular damage clearance, thereby favoring the intracellular accumulation of genomic and mitochondrial damage and subsequent cell death.

## 3 Conclusion and Discussion

The development of acquired chemoresistance to L‐OHP remains the primary cause of treatment failure and poor prognosis in patients with advanced CRC. Identifying novel therapeutic targets and developing innovative drugs with unique MOA represent critical strategies to overcome this clinical bottleneck. In this study, we identified TA, a natural withanolide, as a promising candidate with favorable preclinical tolerability for the treatment of CRC. By systematically deconvoluting its targets, we identified PLK1 as a direct functional target of TA. In parental CRC cells, a lethal dose of TA disrupts PLK1‐p53 binding, leading to p53 nuclear translocation and subsequent activation of ferroptosis [50]. Acquired L‐OHP resistance appears to shift cellular dependency toward a PLK1‐driven vesicular damage‐clearance program. Under this context, a sub‐lethal dose of TA interferes with the PLK1‐CEP55‐ESCRT axis without inducing appreciable mitotic arrest or cytotoxicity, thereby functioning primarily as a chemosensitizer. Indeed, this mechanistic shift is similar to findings with the PLK1 inhibitor BI2536: at sub‐lethal concentrations, it bypasses canonical mitotic arrest and instead specifically attenuates DNA damage repair networks, synergistically enhancing sensitivity to PARP inhibitors [51]. Similar adaptive functional repurposing has been observed for other canonical mitotic regulators. For example, AURKA, a mitotic kinase primarily involved in spindle assembly and chromosome segregation, becomes adaptively activated by TPX2 during chronic EGFR inhibition, where it suppresses apoptosis and supports the emergence of acquired EGFR inhibitor resistance [52]. These observations suggest that canonical mitotic regulators may acquire additional stress‐adaptive functions under therapeutic pressure, providing a broader context for the PLK1–CEP55–ESCRT‐associated mechanism identified here.

Previous studies have demonstrated that PLK1 is aberrantly overexpressed in a variety of human malignancies. PLK1 inhibitors have shown considerable potential in reversing platinum resistance, not only exerting synergistic anticancer effects with L‐OHP but also significantly improving progression‐free survival in patients with platinum‐refractory ovarian cancer and head and neck squamous cell carcinoma [31, 32, 33, 34]. Building upon these findings, we performed a systematic analysis of clinical transcriptomic cohorts to further investigate the potential role of PLK1 in L‐OHP resistance. While previous reports suggested that hyperactivation of PLK1 may regulate the MYC‐CDC7 axis, which drives aberrant cell cycle pathway activation and diminishes the therapeutic efficacy of L‐OHP [32], our study suggests an additional mechanism through which PLK1 may contribute to L‐OHP resistance. We found that resistant CRC cells exhibit a PLK1‐associated damage‐clearance phenotype characterized by increased nuclear membrane budding and MDV formation, which facilitate the removal of chemotherapy‐induced damaged components. These findings further support the clinical relevance of PLK1 in platinum resistance and suggest that targeting PLK1 may represent a promising strategy to overcome chemorefractory CRC.

While protective autophagy has been implicated in chemotherapy resistance [8, 9], our findings suggest an additional early damage‐handling process in L‐OHP‐resistant cells. We observed that, during the early stages of damage, resistant CRC cells undergo physical remodeling of the nuclear and mitochondrial membranes, accompanied by the budding of γ‐H2A.X^+^ micronuclei and the generation of TOM20^−^/PDH^+^ MDVs. These processes may facilitate the removal of damaged nuclear and mitochondrial components while limiting the loss of otherwise functional parental organelles. The TOM20^−^/PDH^+^ phenotype observed here is consistent with selective MDV cargo sorting under oxidative stress, whereby matrix/inner mitochondrial components are packaged while outer membrane proteins are excluded [53, 54]. Such selective packaging, previously linked to PINK1/Parkin‐ and OPA1/SNX9‐associated MDV pathways, may facilitate the removal of locally damaged mitochondrial contents, including matrix components and mtDNA, while preserving the remaining mitochondrial network [54, 55].

Mechanistically, our findings implicate the PLK1–CEP55–ESCRT axis in this vesicle‐associated damage‐clearance process. Classically, PLK1 and CEP55 coordinate midbody abscission during the late stages of cytokinesis. PLK1 phosphorylates CEP55 at Ser436 to prevent its premature interaction with KIF23, ensuring the spatiotemporal recruitment of ESCRT to the midbody [48, 49]. Our data suggest that L‐OHP‐resistant cells may repurpose components of this cytokinetic machinery to facilitate damage handling. Elevated PLK1 activity maintains CEP55 Ser436 phosphorylation, which reduces its association with KIF23 and favors its availability for ESCRT recruitment at nuclear and mitochondrial budding sites. Inhibition of PLK1 by TA, or introduction of a phospho‐dead CEP55^S436A^ mutant, impaired ESCRT recruitment and subsequently suppressed nuclear bud formation and MDV release. These findings suggest an additional, non‐canonical role for the PLK1–CEP55–ESCRT axis in vesicle‐associated damage handling under chemotherapeutic stress.

While TA showed favorable tolerability in the tested preclinical models and enhanced the antitumor efficacy of L‐OHP in vivo, its pharmacokinetic profile remains a limitation to its clinical translatability. Pharmacokinetic studies revealed low oral bioavailability relative to intraperitoneal administration, suggesting substantial presystemic elimination, potentially involving hepatic first‐pass metabolism, a common liability of highly oxygenated natural product scaffolds [56]. To advance TA toward clinical application, structural optimization focusing on metabolically labile sites, or the development of prodrug strategies, may help improve metabolic stability and oral absorption. Alternatively, incorporation into advanced delivery platforms may help circumvent first‐pass metabolism and enhance systemic exposure [57].

In conclusion, our study suggests a previously unappreciated role of the PLK1–CEP55–ESCRT axis in vesicle‐associated damage clearance that may contribute to L‐OHP resistance in CRC. TA targets PLK1 and sensitizes L‐OHP‐resistant CRC cells to L‐OHP by interfering with this damage‐clearance process. These findings provide new insights into the adaptive responses of CRC cells to platinum‐induced stress and support further investigation of the PLK1–CEP55–ESCRT axis and TA as potential strategies for overcoming L‐OHP resistance.

## Author Contributions

Y.Y. was involved in the study design, conducted experiments and drafted the manuscript; J.L. evaluated the activity of CRC organoids; L.W. and Z.F. were responsible for the activity evaluation of CRC organoids; Y.L. synthesized TA PROTACs, TA‐Nap and TA‐DA; H.Z. and Z.W. were responsible for evaluating the anti‐tumor activity of TA against DLD‐1 cell‐derived xenograft tumor models; R.Z., W.X. and S.X. assisted the SPR and MST experiments; Z.Z and D.S. assisted with animal experiments; K.X. and Y.W. extracted and isolated TA from the *Tubocapsicum anomalum*. H.L. and L.C. designed, supervised the study and revised the manuscript. All authors read and approved the final manuscript.

## Funding

We thank the National Natural Science Foundation of China (NSFC, No. U24A20784, 82473812 and 82607360), the China Postdoctoral Science Foundation (No. 2024MD764002), the Postdoctoral Fellowship Program of CPSF (No. GZC20250604), the Fujian Provincial Science and Technology Innovation Joint Fund Project (No. 2024Y9503), “Select the best candidates to lead key research projects” of Fujian University of Traditional Chinese Medicine (No. XJB2022008, XJB2023001), the Foundation of Fujian University of Traditional Chinese Medicine (No. X2023001‐Talent), and Project of Frontier Technology Platform for Research Projects of Liaoning Provincial Department of Education in 2024 (No. LJ232410163056) for financial supports.

## Ethics Statement

Malignant and primary colorectal tissues were collected from patients at Nanjing Hospital of Chinese Medicine affiliated to Nanjing University of Chinese Medicine undergoing surgical procedures at the Department of Colon and Rectal Surgery. All experiments were reviewed and approved by the Ethics Board of Nanjing Hospital of Chinese Medicine and performed under approved protocols. Project: Collaborative R&D of Colorectal Cancer Precision Medicine Platform Based on Colorectal Cancer Organoid Biobank and Early Prevention and Treatment System of Traditional Chinese Medicine (KY2022309). Written informed consent form for research was acquired from donors prior to the sampling procedure. Samples were acquired from adult patients who were treatment‐naive. Pathological analysis of all samples was performed at the Department of Pathology, Nanjing Hospital of Chinese Medicine. All animal experiments were approved by the Institutional Animal Care and Use Committee (IACUC) of Shenyang Pharmaceutical University (SYPU‐IACUC‐S2023‐0927‐106) and Fujian University of Traditional Chinese Medicine (1N2025099, 3W2025121 and 1N2025075).

## Conflicts of Interest

The authors declare no conflicts of interest.

## Supporting information




**Supporting File**: advs78310‐sup‐0001‐SuppMat.docx.

## Data Availability

The data that supports the findings of this study are available in the supplementary material of this article.
