# Supplementary material for: Tubocapsenolide A Overcomes Oxaliplatin Resistance in Colorectal Cancer by Targeting PLK1 to Impair CEP55–ESCRT‐Associated Vesicular Damage Clearance
Source: Adv Sci (Weinh). 2026 Oct 18:e78310. Online ahead of print. doi: 10.1002/advs.78310 (PMC13677074; doi:10.1002/advs.78310)
Supplement: Supplementary file 1 [file ADVS-9999-e78310-s001.docx]

**Supporting Information**

**Tubocapsenolide A Overcomes Oxaliplatin Resistance in Colorectal Cancer by Targeting PLK1 to Impair CEP55–ESCRT-Associated Vesicular Damage Clearance**

Yueying Yang^1,2,#^, Jing Liang^1,2,#^, Lu Wang^3,#^, Hongyan Zhang^4,#^, Zhiruo Zhang^1^, Yang Liu^1,2^, Rui Zhu^1^, Wen Xu^2^, Shaohua Xu^2^, Dejuan Sun^1^, Ke Xiang^2^, Yali Wang^2^, Zhimin Fan^3,*^, Zheng Wang^5,*^, Hua Li^1,2,*^, Lixia Chen^1,2,*^

^1^*Wuya College of Innovation, Shenyang Pharmaceutical University, Shenyang, 110016, People’s Republic of China.*

^2^*Institute of Digital Materia Medica and Structural Pharmacology, Fujian Key Laboratory of Chinese Materia Medica, College of Pharmacy, Fujian University of Traditional Chinese Medicine, Fuzhou, 350122, People’s Republic of China.*

^3^*Jiangsu Clinical Innovation Center for Anorectal Diseases of T.C.M, Nanjing Hospital of Chinese Medicine, Nanjing University of Chinese Medicine, Nanjing, 210022, People’s Republic of China.*

^4^*Research Center for Tissue Engineering and Regenerative Medicine, Union Hospital, Tongji Medical College, Huazhong University of Science and Technology, Wuhan, 430022, People’s Republic of China.*

^5^*Department of Gastrointestinal Surgery, Union Hospital, Tongji Medical College, Huazhong University of Science and Technology, Wuhan, 430022, People’s Republic of China.*

^#^The authors have contributed equally to this work.

*Correspondence and requests for materials should be addressed to Lixia Chen (syzyclx@163.com), Hua Li (lihua@fjtcm.edu.cn), Zheng Wang (zhengwang@hust.edu.cn), Zhimin Fan (fanzm@njucm.edu.cn).

**Figure S1. TA exhibits broad-spectrum anti-tumor activity and favorable tolerability in experimental models.** (A) Dose-response curves illustrating the *in vitro* inhibition rates of TA against a panel of human CRC cell lines (SW480, HCT116, DLD-1, HT-29 and Caco-2). Data are shown as the mean ± s.d. (n = 3). (B) Schematic representation of the establishment of the DLD-1 subcutaneous xenograft mouse model and the corresponding TA treatment regimen (10 mg/kg, i.g.). (C) Tumor growth curves depicting the dynamic changes in tumor volume (left), representative macroscopic images of excised xenograft tumors (middle), and a bar graph of terminal tumor weights (right) from the DLD-1 model (n = 6). (D) Dynamic monitoring of mouse body weights in the DLD-1 xenograft model throughout the 19-day treatment period (n = 6). (E) Visceral organ indices (ratio of organ weight to body weight) for the heart, liver, spleen, lung, and kidney in healthy male and female mice subjected to safety evaluations. Data are shown as the mean ± s.d. (n =3). Statistical analysis was performed using one-way ANOVA followed by Tukey’s multiple comparisons test. ns, not significant. (F) Serum biochemical evaluation of cardiac (CK, CK-MB), hepatic (ALT, AST), and renal (UA, UREA) functional markers in DLD-1 tumor-bearing mice (n = 6). (G) H&E staining images of major organs harvested from tumor-bearing mice treated with vehicle, TA or L-OHP. Scale bar, 250 μm. (H) H&E staining images of major organs treated with vehicle or TA. Scale bar, 50 μm. (C, D, F) Data are shown as the mean ± s.d. Statistical analysis was determined using a two-tailed unpaired Student’s t-test. *p < 0.05, **p < 0.01; ns, not significant.

**Figure S2. Identification and validation of PLK1 as the direct target of TA.** (A) The effect of TA-V, TA-P, TA-N and TA-B on the cell viability of HCT116 cells after 24 h treatment was determined by CCK8. Data are presented as mean ± s.d. (n = 3). (B) Immunoblot analysis of the degradation of PLK1 in HCT116 cells after treated with indicated concentrations TA-N for 24 h. (C) Western blot for PLKs expression in SW480 and DLD-1 cells treated with TA-N for 24 h. (D) Immunoblot analysis of PLK1 degradation in HCT116 cells after treatment with TA or Nutlin-3 for 24 h. (E) Immunoblot analysis of time-dependent degradation of PLK1 in HCT116 cells after treatment with 4 μM TA-N for 3-48 h. (F) Immunoblot analysis of PLK1 in HCT116, SW480 and DLD-1 cells pretreated with TA, MG132, or Nutlin‑3 for 4 h, followed by treatment with DMSO or TA‑N for 24 h. (G) Ubiquitination assays evaluating TA-N-induced PLK1 polyubiquitination. Left: Dose-dependent polyubiquitination of exogenously expressed HA-PLK1 following TA-N treatment (0, 2, and 4 μM). Right: Time-dependent polyubiquitination of endogenous PLK1 upon TA-N treatment (0, 6, and 12 h). (H) Competitive ubiquitination assay confirming the target-PROTAC-E3 ternary complex-dependent mechanism. HCT116 cells were pretreated with 4 μM TA or 16 μM Nutlin-3 for 4 h, followed by DMSO or TA-N treatment for 24 h, and PLK1 ubiquitination levels were assessed by immunoblot. All experiments (G, H) were conducted in the presence of 0.5 μM MG132 to accumulate ubiquitinated substrates. WCL, whole cell lysate. (I) HCT116 cells were treated with 4 μM TA or DMSO vehicle for 3 h. Cell lysates were then heated across a temperature range of 55-65°C, and the remaining soluble PLK1 protein levels were analyzed by immunoblotting. GAPDH served as a loading control. Data are presented as mean ± s.d. (n = 3). Statistical significance was determined by two-way ANOVA followed by Šídák’s multiple comparisons test. Exact p-values are indicated in the figures. (J) HCT116 cell lysates were pre-incubated with vehicle or increasing concentrations of TA (1, 2, or 4 μM) for 3 h, followed by digestion with pronase (or no pronase control) for 30 min at 37°C. PLK1 protein levels were analyzed by immunoblotting. Quantification of PLK1 band intensity normalized to the vehicle control without pronase is shown in the lower panel. Data are presented as mean ± s.d. (n = 3). Statistical significance was determined by one-way ANOVA followed by Dunnett’s multiple comparisons test. Exact p-values are indicated in the figures. (K) MST analysis confirming the K_d_ of TA for full-length PLK1, PLK1-CD, and PLK1-PBD, consistent with the SPR results. Data are shown as the mean ± s.d. (n = 3).

**Figure S3. Validation of TA-induced PLK1-dependent cytotoxicity.** (A) Cell viability of DLD-1 and PLK1^KD^ cells treated with 1 μM TA or DMSO for 24 h. Data are presented as mean ± s.d. (n = 6). Statistical significance was determined by two-way ANOVA followed by Šídák’s multiple comparisons test. (B) Colony formation assays of HCT116 and DLD-1 cells showing the rescue effect of PLK1^KD^ against TA. (C) Automated capillary Western blot analysis of ferroptosis-related proteins in T362 patient-derived CRC organoids treated with 0, 20, 80, and 320 μM TA for 24 h. Protein expression levels were determined via chemiluminescence and normalized to area under the curve. The corresponding signal peak data and quantified areas are displayed in **Fig. S4B** and **Table S3**. (D) Immunoblot validation of ferroptosis markers *in vivo* using tumor tissues from Vehicle, TA(L), and TA(H) xenograft models. (E) HCT116 and PLK1^KD^ cells were treated with 2 μM TA or DMSO for 24 h. Ferroptosis-related proteins were analyzed by Western blot. (F-H) Immunoblot of ferroptosis markers (F), and fluorescence quantification of FerroOrange (G) and Liperfluo (H) in HCT116 cells treated with TA alone or in combination with 2 μM Fer-1 (n = 3). Data are presented as mean ± s.d. Statistical analysis was performed using one-way ANOVA followed by Tukey’s multiple comparisons test. Exact p-values are indicated in the figures.

**Figure S4.** **Raw signal peak data generated by the Jess automated capillary Western blot system.** (A, B) The peaks represent the chemiluminescent intensity of target proteins (ACSL4, GPX4, Nrf2, HO-1, and β-Actin) corresponding to the virtual pseudo-blots shown in **Fig. 3O** and **Fig. S3C**.

**Figure S5. TA synergizes with L-OHP to overcome chemoresistance.** (A) Quantification of PLK1 expression in HCT116-derived and HCT116-OR-derived xenograft tumor tissues (n = 3). Statistical analysis was determined using a two-tailed unpaired Student’s t-test between the indicated group and the vehicle group. (B) HCT116-OR cells treated with increasing concentrations of TA for 24 h, after which cell viability was assessed (n = 3). (C) The synergistic interaction between TA and L-OHP was evaluated using the Loewe and Bliss model in SynergyFinder. Synergy scores above 10 are generally considered synergistic. (D) Dose–response curves of HCT-8 and HCT-8-OR cells to L-OHP and of HCT-8-OR cells to TA. The effect of 0.2 μM TA on L-OHP sensitivity in HCT-8-OR cells was also assessed (n = 3). (E, F) Cell viability of HCT116-OR and PLK1^KD^ HCT116-OR (E) and HCT-8-OR and PLK1^KD^ HCT-8-OR (F) cells following treatment with 0.2 μM TA and 10 μM L-OHP, with or without HA-PLK1 re-expression, as indicated for 24 h (n = 3). (G) Cell viability of normal human colonic epithelial NCM460 cells following treatment with the indicated concentrations of TA for 24 h (n = 3), ****p < 0.0001. (H, I) Tumor growth curves depicting tumor volume over time (H) and box plots of final tumor weights (I) across the indicated treatment groups. (J) Body weight changes in HCT116-OR xenograft-bearing mice (n = 9). Data are presented as mean ± s.d. Statistical analysis was performed using one-way ANOVA followed by Tukey’s multiple comparisons test. Exact p-values are indicated in the figures.

**Figure S6. Rigorous batch effect correction of the integrated multi-cohort CRC transcriptomic datasets.** (A, B) Boxplots displaying the distribution of gene expression levels across the seven independent GEO CRC cohorts (GSE104645, GSE19860, GSE39582, GSE81653, GSE106584, GSE28702, and GSE72970) before (A) and after (B) batch effect correction using the ComBat algorithm. (C, D) Density plots illustrating the overall gene expression distributions across the respective datasets before (C) and after (D) batch effect removal, demonstrating the successful alignment of expression profiles. (E, F) Principal Component Analysis (PCA) score plots mapping the sample distributions of the seven cohorts before (E) and after (F) correction. The distinct, dataset-dependent clustering observed prior to correction (E) is effectively eliminated, resulting in a harmonized, biologically driven sample distribution (F).

**Figure S7.** **PLK1 functions as a regulator of PLK1-CEP55-ESCRT-dependent vesicular clearance that contributes to L-OHP resistance in CRC.** (A) ROC curve analyses of the representative proliferation markers MKI67 and PCNA for distinguishing Non-Responders from Responders in the integrated clinical cohort (n = 408). AUC values and 95% confidence intervals are indicated in the graphs. (B, C) Statistical validation of the OPLS-DA model by permutation test. A 100-time permutation test was conducted to evaluate the reliability of the OPLS-DA model. (D) Correlation analysis of enriched biological pathways. The heatmap illustrates the pairwise Pearson correlation coefficients between various biological processes and cellular components. The color scale indicates the degree of correlation, with dark purple representing a strong positive correlation and light yellow representing a strong negative correlation. The size of the squares corresponds to the absolute value of the correlation coefficient. (E) Distribution of relative γ-H2A.X MFI in the main nucleus. (F) Quantification of relative DNA levels in micronuclei in HCT-8 and HCT-8-OR cells following treatment with 5 μM L-OHP for 6 h. Where indicated, HCT-8-OR cells were co-treated with 0.2 μM TA (n = 3). (G) Immunofluorescence of TOM20⁻/PDH⁺ MDVs. Scale bar, 5 μm. (H) Relative *mtND1* copy numbers quantified by qPCR in subcellular vesicular fractions isolated by ultracentrifugation and verified after treatment with L-OHP for 6 h (n = 3). (E, G, H) HCT116, PLK1^OE^ HCT116, HCT116-OR, and PLK1^KD^ HCT116-OR cells were treated with 10 μM L-OHP for 3 h, with 0.5 μM TA co-treatment as indicated. (I) Representative immunofluorescence images of TOM20 and PDH, along with quantification of TOM20⁻/PDH⁺ MDVs in HCT-8 and HCT-8-OR cells. (J) qPCR quantification of *mtCo3*, *mtD-loop*, and *mtND1* levels in MDV fractions. All groups were treated with 5 μM L-OHP for 6 h, with HCT-8-OR cells additionally co-treated with 0.2 μM TA as indicated (n = 3). Data are presented as mean ± s.d. Statistical analysis was performed using one-way ANOVA followed by Tukey’s multiple comparisons test. Exact p-values are indicated in the figures.

**Figure S8. Validation of the PLK1CEP55‑ESCRT axis driven by L‑OHP‑resistant cells.** (A) Bubble and Sankey plot illustrating the significant enrichment of ESCRT, nuclear envelope budding (NEB), and MDV-related pathways following TA treatment. (B) Immunofluorescence images showing the enrichment of the ESCRT-III subunit CHMP4B on mitochondria in HCT116-OR cells treated with 10 μM L-OHP for 3 h. Scale bar, 5 μm. (C) Representative immunofluorescence images showing CHMP4B recruitment to nuclear budding sites in HCT-8 and HCT-8-OR cells. Scale bar, 2.5 μm. (D) Representative immunofluorescence images showing CHMP4B recruitment to mitochondria in HCT-8 and HCT-8-OR cells. All groups were treated with 5 μM L-OHP for 3 h, with HCT-8-OR cells additionally co-treated with 0.2 μM TA as indicated. Scale bars are indicated in the images. (E) Supplementary scatter plot confirming the robust positive correlation between PLK1 and CEP55 expression. (F) Dose-dependent inhibition of CEP55 Ser436 phosphorylation by TA in HCT116-OR cells following 12 h treatment. Levels of p-CEP55, total CEP55, and GAPDH were determined by Western blotting. (G) Western blot analysis of p-CEP55, total CEP55, and PLK1 expression in HCT116, PLK1^OE^ HCT116, HCT116-OR, PLK1^KD^ HCT116-OR, and TA-treated HCT116-OR cells, as indicated. All groups were treated with 10 μM L-OHP for 12 h. (H) Western blot analysis of p-CEP55, total CEP55, and GAPDH in HCT-8 and HCT-8-OR cells treated with 5 μM L-OHP for 12 h, with 0.2 μM TA co-treatment as indicated. (I) *In vitro* phosphorylation of CEP55-WT and CEP55-S436A peptides by recombinant PLK1, determined using the ADP-Glo kinase assay. PLKtide served as a positive-control substrate (n = 3). (J) Concentration-dependent inhibition of PLK1 kinase activity by TA using CEP55-WT as the substrate at 50 or 200 μM ATP. IC₅₀ values were determined by nonlinear regression (n = 3). (K) HCT116 and HCT116-OR cells were treated with 10 μM L-OHP for 12 h, with HCT116-OR cells additionally treated with 0.2 μM TA as indicated. Co-IP analysis was performed to evaluate the association between TSG101 and p-CEP55. Data are presented as mean ± s.d. Statistical analysis was performed using one-way ANOVA followed by Tukey’s multiple comparisons test. Exact p-values are indicated in the figures.

**Figure S9.** **CEP55 Ser436 phosphorylation promotes ESCRT recruitment and vesicle-mediated damage extrusion.** CEP55^KD^ HCT116-OR cells were rescued with HA-CEP55^WT^, HA-CEP55^S436A^, or HA-CEP55^S436D^ as indicated. Cells were treated with 10 μM L-OHP for 6 h for qPCR analyses (A, C) or for 3 h for immunofluorescence analyses (B, D, E). (A) Quantification of relative DNA levels in micronuclei. (B) Representative immunofluorescence images of TOM20 and PDH showing TOM20⁻/PDH⁺ MDVs. (C) qPCR quantification of *mtCo3*, *mtND1*, and *mtD-loop* levels in isolated MDV fractions (n = 3). (D) Representative immunofluorescence images showing CHMP4B recruitment to mitochondria (n = 3). (E) Representative immunofluorescence images showing CHMP4B recruitment to nuclear budding sites. Data are presented as mean ± s.d. Statistical analysis was performed using one-way ANOVA followed by Tukey’s multiple comparisons test. Exact p-values are indicated in the figures. Scale bars are indicated in the images.

**Figure S10. PLK1-CEP55 signaling maintains nuclear envelope integrity in L-OHP-resistant cells.** (A) Representative confocal images of nuclear envelope morphology visualized by Lamin A/C (red) and DAPI (blue) staining in HCT116-OR, PLK1^KD^ HCT116-OR, and CEP55^KD^ HCT116-OR cells rescued with HA-CEP55 or HA-CEP55^S436A^, following treatment with 10 μM L-OHP for 4 h, with 0.5 μM TA co-treatment as indicated. Scale bar, 5 μm. (B) Quantification of mitochondrial aspect ratio (major/minor axis length) reflecting mitochondrial fragmentation. Higher values indicate elongated mitochondria, while lower values indicate increased fragmentation (n ≥ 25). (C) Western blot analysis of LC3B and p62 in HCT116-OR cells treated with DMSO, 0.5 μM TA, or 0.5 μM TA in combination with 10 μM L-OHP for 3 h in the presence of 100 nM bafilomycin A1 (BafA1). (D) Flow cytometric analysis and quantification of lipid peroxidation using Liperfluo in HCT116-OR cells. (E) Flow cytometric analysis and quantification of intracellular Fe²⁺ levels using FerroOrange. (F) Representative flow cytometric analysis and quantification of cell-cycle distribution by EdU/DAPI staining. (G) Western blot analysis of Bax and Bcl-2 expression. (H) Representative flow cytometric analysis and quantification of apoptosis by Annexin V/PI staining. For (D-H), HCT116-OR cells were treated with DMSO, 0.2 μM TA, or 0.2 μM TA in combination with 10 μM L-OHP for 24 h. Data are presented as mean ± s.d. (n = 3). Statistical analysis was performed using one-way ANOVA followed by Tukey’s multiple comparisons test. Exact p-values are indicated in the figures.

**Table S1.** Pharmacokinetic parameters of TA

|  | TA | | |
| --- | --- | --- | --- |
|  | *i.v.* (0.5 mg/kg) | *i*.*p*. (2.5 mg/kg) | *i*.*g*. (25 mg/kg) |
| T1/2 (h) | 1.574 ± 0.478 | 2.359 ± 0.835 | 3.947 ± 1.843 |
| Tmax (h) | 0.167 | 1.25 ± 0.274 | 1.167 ± 0.258 |
| MRT0-t (h) | 1.324 ± 0.394 | 3.432 ± 0.118 | 4.584 ± 0.494 |
| C0 (μg·L-1) | 4.261 ± 2.661 | 3.039 ± 2.736 | 1.416 ± 1.451 |
| Cmax (μg·L-1) | 57.936 ± 15.329 | 75.06 ± 10.271 | 197.145 ± 38.12 |
| AUC0-t (μg·L-1*h) | 66.556 ± 10.875 | 310.29 ± 46.402 | 638.725 ± 47.949 |
| AUC0-∞ (μg·L-1*h) | 76.44 ± 12.163 | 323.372 ± 58.812 | 650.001 ± 46.837 |
| Vd/F (L·Kg-1) | 14.719 ± 2.854 | 25.862 ± 5.143 | 219.311 ± 100.565 |
| CL/F (L·Kg-1·h-1) | 6.687 ± 1.11 | 7.947 ± 1.425 | 38.624 ± 2.714 |

**Table S2.** Quantified automated capillary Western blot peak areas of T328 organoids

| Sample | β-Actin | GPX4 | ACSL4 | β-Actin | Nrf2 | β-Actin | HO-1 |
| --- | --- | --- | --- | --- | --- | --- | --- |
| T328_0 μM | 279386 | 355524.8 | 3438485 | 166920 | 61347.9 | 316005 | 12968.6 |
| T328_0 μM | 301168 | 361401.2 | 3281506.8 | 161068.2 | 48392.5 | 363678.5 | 12293.3 |
| T328_0 μM | 276510.6 | 376364.3 | 3364194.3 | 172087.1 | 58357.8 | 314952.9 | 11864.3 |
| T328_10 μM | 276819.2 | 252424.1 | 3283330.5 | 115286.2 | 80264.4 | 343461.3 | 21680.9 |
| T328_10 μM | 292879.6 | 251780.8 | 2893925.8 | 104162 | 79939.7 | 350406.4 | 20745.2 |
| T328_10 μM | 305820.1 | 249724.6 | 3265216.5 | 113617 | 72933.8 | 339849 | 27136.4 |
| T328_40 μM | 285153.4 | 177271.1 | 2841133.3 | 113287 | 126301 | 325143.1 | 34074.1 |
| T328_40 μM | 287980.9 | 187858.6 | 2972497.2 | 144253.8 | 118960.6 | 368159.1 | 39450 |
| T328_40 μM | 265311.2 | 167273.4 | 2900965.1 | 147999.4 | 133324.6 | 357928.3 | 37684.3 |

**Table S3.** Quantified automated capillary Western blot peak areas of T362 organoids

| Sample | β-Actin | ACSL4 | GPX4 | β-Actin | Nrf2 | β-Actin | HO-1 |
| --- | --- | --- | --- | --- | --- | --- | --- |
| T362_0 μM | 106350.9 | 1664530.3 | 1015916.5 | 204922.5 | 87553.2 | 327533.9 | 21449.3 |
| T362_0 μM | 89230.5 | 1603975.4 | 797847.7 | 189337.5 | 78672.2 | 356691.6 | 31279.7 |
| T362_0 μM | 113654.8 | 1743701.9 | 957242.4 | 211398.8 | 103943.6 | 344667.7 | 27448.1 |
| T362_20 μM | 88134.7 | 1722224.3 | 523144.2 | 199987.7 | 96353.2 | 312140.9 | 32504.8 |
| T362_20 μM | 72562.3 | 1586125 | 457697.6 | 173151.5 | 90280.1 | 331340.3 | 33838.5 |
| T362_20 μM | 102625.8 | 1663289.2 | 554165.6 | 217049.3 | 95184.5 | 294995.3 | 33170.5 |
| T362_80 μM | 82402.2 | 1620153.3 | 412084.7 | 220453.5 | 133792.6 | 385127.3 | 48431.2 |
| T362_80 μM | 108728.6 | 1798747.2 | 449013.1 | 221588.8 | 132713.3 | 294188 | 39430.2 |
| T362_80 μM | 106960 | 1778169 | 418392.5 | 222401.2 | 134740.8 | 327192.1 | 46197.5 |
| T362_320 μM | 101662 | 1582433.1 | 448727 | 217122.5 | 149820.2 | 380296.8 | 63452.5 |
| T362_320 μM | 90291.7 | 1638030.2 | 349727.5 | 234031.6 | 156645.2 | 366471.2 | 51578.8 |
| T362_320 μM | 65686.4 | 1401258.2 | 270179 | 219367.9 | 157951 | 280391.4 | 51518.7 |

**Table S4.** Antibodies with their respective dilution ratios

| Antibody | Source | Application | Dilution |
| --- | --- | --- | --- |
| PLK1 | Proteintech (#10305-1-AP) | Western blot | 1:4000 |
|  |  | Immunohistochemistry | 1:100 |
|  |  | Immunoprecipitation | 1 μg for 1 mg protein lysate |
|  |  | capillary Western blot | 1:15 |
| PLK2 | Proteintech (#15956-1-AP) | Western blot | 1:4000 |
| PLK3 | Proteintech (#10977-1-AP) | Western blot | 1:2000 |
| PLK4 | Proteintech (#8750-1-AP) | Western blot | 1:500 |
| β-Actin | Proteintech (#66009-1-Ig) | Western blot | 1:5000 |
| GAPDH | Proteintech (#60004-1-Ig) | Western blot | 1:5000 |
| HA | Proteintech (#51064-2-AP) | Western blot | 1:5000 |
|  |  | Immunoprecipitation | 1 μg for 1 mg protein lysate |
|  |  | Immunofluorescence | 1:500 |
| His | Proteintech (#66005-1-Ig) | Western blot | 1:5000 |
| GPX4 | Cell Signaling Technology (#52455) | Western blot | 1:1000 |
|  |  | capillary Western blot | 1:10 |
| ACSL4 | Proteintech (#22401-1-AP) | Western blot | 1:4000 |
|  |  | capillary Western blot | 1:500 |
| Nrf2 | Cell Signaling Technology (#12721) | Western blot | 1:1000 |
|  |  | capillary Western blot | 1:10 |
| HO-1 | Proteintech (#10701-1-AP) | Western blot | 1:5000 |
|  | Cell Signaling Technology (#82206) | capillary Western blot | 1:10 |
| Ubiquitin | Cell Signaling Technology (#3936) | Western blot | 1:1000 |
| CEP55 | Proteintech (#23891-1-AP) | Western blot | 1:1000 |
| ALIX | Proteintech (#12422-1-AP) | Western blot | 1:5000 |
|  |  | Immunoprecipitation | 2 μg for 1 mg protein lysate |
| KIF23 | Proteintech (#28587-1-AP) | Western blot | 1:2000 |
|  |  | Immunoprecipitation | 2 μg for 1 mg protein lysate |
| γ-H2A.X | Cell Signaling Technology (#2577) | Immunofluorescence | 1:500 |
|  |  | Immunohistochemistry | 1:100 |
| 53BP1 | Cell Signaling Technology (#4937) | Immunofluorescence | 1:500 |
| TOM20 | Proteintech (#11802-1-AP) | Immunofluorescence | 1:1000 |
| PDH | Proteintech (#21176-1-AP) | Immunofluorescence | 1:1000 |
| CHMP4B | Proteintech (#13683-1-AP) | Immunofluorescence | 1:200 |
| Ki67 | Proteintech (#27309-1-AP) | Immunohistochemistry | 1:5000 |
| TSG101 | Proteintech (#28283-1-AP) | Western blot  Immunoprecipitation | 1:2000  1 μg for 1 mg protein lysate |
| LC3B | Abclonal (#A7198) | Western blot | 1:2000 |
| p62 | Abclonal (#A19700) | Western blot | 1:4000 |
| Bax | Abclonal (#A19684) | Western blot | 1:1000 |
| Bcl2 | ABclonal (#A19693) | Western blot | 1:1000 |

**Table S5.** Primers for qPCR

| Gene | Forward primer | Reverse primer |
| --- | --- | --- |
| *Alu* | 5'-CATGGTGAAACCCCGTCTCTA-3' | 5'-GCCTCAGCCTCCCGAGTAG-3' |
| *mtCo3* | 5'-ATGACCCACCAATCACATGC-3' | 5'-ATCACATGGCTAGGCCGGAG-3' |
| *mtD-loop* | 5'-TTAACTCCACCATTAGCACC-3' | 5'-GAGGATGGTGGTCAAGGGAC-3' |
| *mtND1* | 5'-ATGGCCAACCTCCTACTCCTC-3' | 5'-CTACAACGTTGGGGCCTTT-3' |
| *PLK1* | 5'-CACCAGCACGTCGTAGGATTC-3' | 5'-CCGTAGGTCATCGTCATTTCTC-3' |
| *GAPDH* | 5'-GGAGCGAGATCCCTCCAAAAT-3' | 5'-GGCTGTTGTCATACTTCTCATGG-3' |

**Scheme S1.** Synthesis of the TA-Biotin Probe

**Scheme S2.** Synthesis of the TA-Nap fluorescent Probe

**Scheme S3.** Synthesis of the TA-DA photo-crosslinkable Probe

**Methods**

**Cell lines and culture**

Human colorectal cancer cell lines (HCT116, DLD-1, SW480, HT-29, and Caco-2) were obtained from the American Type Culture Collection (ATCC) or the Cell Bank of the Chinese Academy of Sciences. NCM460, HCT-8, HCT-8-OR, and HCT116-OR cells were obtained from Shanghai Zhong Qiao Xin Zhou Biotechnology (China). All lines were authenticated by short tandem repeat (STR) profiling and routinely tested for mycoplasma contamination. Cells were maintained in McCoy’s 5A, RPMI-1640 or DMEM supplemented with 10% fetal bovine serum (FBS), 100 U/mL penicillin, and 100 μg/mL streptomycin at 37°C in a humidified 5% CO₂ incubator.

***In vitro* cell viability assay**

Cell Counting Kit-8 (CCK8) method was performed to measure cell survival. HCT116, HCT116-OR, DLD-1, SW480, HT-29, Caco-2, NCM460, HCT-8, or HCT-8-OR (0.5×10^4^ cells/well) were seeded in 96-well plates in a total volume of 80 μL of media for 24 h. Serially diluted compounds in 20 μL of media were added to the cells. After 24 h, 10 μL CCK8 (ApexBio Technology, #K1018) was added and incubated at 37°C. The OD value was measured by Multimode reader (Thermo Fisher, Varioskan LUX) at 450 nm. The estimated in vitro IC_50_ values were calculated by using GraphPad Prism 9.0.

**PDO culture and drug sensitivity assay**

CRC organoids were digested and seeded in 384-well plates with a density of 500 cells/well. After 24 h, PDOs were exposed to a concentration gradient of TA, L-OHP, or 5-FU for 3 days. Bright-field morphological images were captured using an inverted microscope to document architectural disintegration. Cell viability of the organoids was quantified utilizing the CellTiter-Glo® 3D Cell Viability Assay (Promega), measuring the luminescent signal proportional to the ATP content.

***In vivo* xenograft tumor models**

Male BALB/c-nude mice (6-8 weeks old, 18-22 g) were purchased from Beijing Huafukang Biological Technology Co. Ltd. and housed under specific pathogen-free conditions. To establish the subcutaneous xenograft models, **HCT116 cells (5×10⁶ cells/mouse), DLD-1 cells (4×10⁶ cells/mouse), or HCT116**-OR **cells (2×10⁶ cells/mouse) suspended in PBS mixture were subcutaneously injected into the axilla of male BALB/c nude mice**. When the tumor volume reached approximately 50 mm³, the mice were randomly assigned to different treatment groups. Mice were randomly assigned to treatment groups, but tumor measurements were not performed under formal blinding. Histological analyses were conducted with treatment-group information concealed during image acquisition and quantitative analysis. For the HCT116 model, mice received Vehicle, TA (20 or 40 mg/kg, i.g., once daily), or L-OHP (10 mg/kg, i.p., every 3 days) for 18 days. For the DLD-1 model, mice were treated with Vehicle or TA (10 mg/kg, i.g., once daily) for 13 days. For the HCT116-OR model, mice were treated with Vehicle, L-OHP (10 mg/kg, i.p., twice a week), FOLFOX regimen (10 mg/kg L-OHP + 50 mg/kg 5-FU + 100 mg/kg LV, i.p., twice a week), TA (10 mg/kg, i.p., once every two days)**, and** L-OHP + TA combination (10 mg/kg L-OHP, i.p., twice a week + 1 mg/kg TA, i.g., once every two days). The tumor volume V was determined every day by measuring the **length (L, mm), width (W, mm) and height (H, mm)** of the tumors and using the formula V = π/6 × L × W × H to calculate the volume (mm³), and the body weight was recorded every day. At the experimental endpoint, mice were euthanized, and the tumors and major visceral organs were excised, weighed, and processed for further histological analysis.

**Pharmacokinetic profiling**

To evaluate the pharmacokinetic properties of TA, healthy Wistar rats were randomly assigned into three groups (n = 7 per group) and administered a single dose of TA via intravenous (i.v., 0.5 mg/kg), intraperitoneal (i.p., 2.5 mg/kg), or intragastric (i.g., 25 mg/kg) routes. Blood samples were collected from the jugular vein into heparinized tubes at designated time points over a 24 h period. Plasma was immediately separated by centrifugation and stored at -80°C until analysis. Plasma concentrations of TA were quantitatively determined using a validated liquid chromatography-tandem mass spectrometry (LC-MS/MS) method. Pharmacokinetic parameters were calculated using non-compartmental analysis.

**Acute toxicity evaluation**

For acute toxicity assessment, a sequential dose-escalation design was employed. Healthy male and female BALB/c mice were randomly assigned to vehicle and TA groups (n = 6 per group). Mice in the TA group received sequential oral doses of TA at 125, 250, 500, and 1000 mg/kg, with an observation interval of 2 days between consecutive dose levels, whereas control mice received vehicle according to the same schedule. At the experimental endpoint, terminal blood samples were collected following eyeball enucleation. Serum was obtained by centrifugation of coagulated blood samples at 5,000 rpm for 10 min, whereas plasma was prepared from blood collected into heparinized tubes and centrifuged under the same conditions. Blood biochemical parameters were measured using commercial assay kits (Nanjing Jiancheng Bioengineering Institute, China) according to the manufacturer’s instructions. These included markers of liver function (ALT, AST, ALP, TP, ALB, and T-Bil), renal function (Cr, UA, BUN, and UREA), cardiac injury (CK and CK-MB), and total cholesterol (T-CHO). Visceral organ indices were calculated as the ratio of organ weight to body weight and expressed as percentages.

**H&E staining**

Major vital organs (heart, liver, spleen, lung, and kidneys) harvested from both healthy and tumor-bearing mice were immediately fixed in 4% paraformaldehyde for 24 h. The tissues were then dehydrated through a graded ethanol series, embedded in paraffin, and sectioned at a thickness of 4-5 μm. The sections were stained with H&E according to standard histological protocols. The stained slides were evaluated and imaged under a light microscope to assess any signs of morphological lesions, necrosis, or inflammatory cell infiltration.

**Mass spectrometry for proteomics**

HCT116 cells were treated with DMSO, 1 μM TA, 1 μM TA-N, or 1 μM TA-P for 24 h. After treatment, the cells were collected and resuspended in lysis buffer containing 8 M urea, 2 mM EDTA, 10 mM DTT, and 1% protease inhibitor cocktail. The protein concentration was determined using the BCA assay. Proteins were then precipitated by adding cold 20% trichloroacetic acid (TCA) and incubating at 4°C for 2 h. Following centrifugation at 4,500×g for 5 min, the protein pellets were washed three times with cold acetone. The precipitated proteins were resuspended in 200 mM triethylammonium bicarbonate (TEAB) buffer and digested with trypsin at a 1:50 (w/w) enzyme-to-protein ratio overnight. After trypsin digestion, the resulting peptides were desalted using Strata X columns (Phenomenex) and vacuum-dried. The dried peptides were dissolved in 0.5 M TEAB and labeled according to the manufacturer’s instructions for the 6-plex Tandem Mass Tag (TMT) kit. The labeled peptides were separated using a reversed-phase analytical column on an EASY-nLC 1000 UPLC system (Thermo Scientific). The separation was performed using a gradient of buffer A (0.1% formic acid and 2% acetonitrile in water) and buffer B (0.1% formic acid and 90% acetonitrile). The separated peptides were then subjected to nanospray ionization (NSI) and analyzed by tandem mass spectrometry (MS/MS) using a Q Exactive Plus mass spectrometer (Thermo Scientific). Intact peptides and their secondary fragments were detected and analyzed using a high-resolution Orbitrap mass analyzer. The electrospray voltage was set to 2.0 kV. The MS/MS data were processed using the Mascot search engine (version 2.3.0) and matched against the appropriate proteome database. Trypsin/P was specified as the cleavage enzyme, allowing up to two missed cleavages and a minimum peptide length of seven amino acid residues. The mass tolerance was set to 10 ppm for precursor ions and to 0.02 Da for fragment ions. Cysteine alkylation was set as a fixed modification, and methionine oxidation was set as a variable modification. The data were filtered at a 1% false discovery rate (FDR). Proteins with an absolute fold change of ≥1.5 were considered significantly altered. Sample processing and data analysis were performed by Jingjie PTM Biolabs Inc. (Hangzhou, China).

**Immunoblot** **assay**

Following the designated treatments, cells were lysed in RIPA buffer and sonicated. After centrifugation (12,000×g, 10 min, 4°C), the protein concentrations of the supernatants were quantified using a BCA kit (Beyotime, #P0012). Equal amounts of total protein extracts were resolved by 6-15% SDS-PAGE and transferred to PVDF membranes (Millipore, #IPVH00010). The membranes were blocked with 5% non-fat milk, probed with primary antibodies overnight at 4°C, and subsequently incubated with HRP-conjugated secondary antibodies. Immunoreactive bands were visualized using ECL reagents (Meilunbio, #MA0186) and detected on a Tanon-5200 imaging system (Tanon, China). A complete list of antibodies with their respective dilution ratios is summarized in **Table S4**.

**Co-immunoprecipitation analysis**

After treatment with compounds for the designated durations, the harvested cells were lysed in IP lysis buffer (Beyotime, # P0013) and subjected to sonication. The lysates were centrifuged at 12,000×g for 10 min at 4°C to remove insoluble debris. Protein concentrations of the clarified supernatants were determined, and equal amounts of total protein were incubated with specific primary antibodies overnight at 4°C with gentle rotation. Subsequently, 20 μL of Protein A+G Agarose beads (Beyotime, #P2055) were added to each sample and incubated for an additional 4 h at 4°C. The immunoprecipitated complexes were collected by centrifugation, washed three times with ice-cold PBS, and eluted by boiling in 1× SDS loading buffer for 5 min. Finally, the immunoprecipitated samples, along with the whole-cell lysates serving as Input controls, were subjected to SDS-PAGE and analyzed by immunoblotting. A complete list of antibodies with their respective dilution ratios is summarized in **Table S4**.

**Automated capillary Western blot analysis**

1 mg of colon tissue was added to 30 μL of IP lysis buffer (Beyotime, #P0013) and homogenized using the MP FastPrep-24 5G™ Sample Preparation System (6.0 m/s) with 1.4 mm ceramic spheres (Thermo Scientific, #MP116913100) for 45 s. The homogenates were left on ice for 10 min to allow the froth to settle, then centrifuged at 13,000 g for 15 min at 4°C. The concentration of soluble protein was determined using the BCA Protein Assay kit (Beyotime, #P0012). Samples were stored at -80°C before use. The samples were denatured with 5× Fluorescent Master Mix (ProteinSimple, a Bio-Techne brand, #PS-ST01EZ-8) with final concentrations of 1% SDS and 40 mM DTT, followed by incubation at 95°C for 5 min and centrifugation at 1000 rpm for 5 min. Protein expression was analyzed using the automated capillary Western blot system, Jess (ProteinSimple), following the manufacturer’s standard protocol [1-3]. 5 μL biotinylated ladder and 3 μL of the denatured sample were loaded into row A of the 12-230 kDa pre-filled plate (ProteinSimple, #SM-W004-1). Rows B to F were filled with antibody diluent, primary antibody, streptavidin‑HRP, goat anti‑rabbit secondary antibody, and a luminol‑peroxide solution, respectively (ProteinSimple, #DM-001). The loaded plates were centrifuged at 2500 rpm for 5 min to remove bubbles from the wells. The plate and capillaries were loaded into the Jess equipment, and the default settings were used to run the chemiluminescence‑based program for immunodetection of the target protein. After each run, electropherograms generated by Compass software for each capillary were analyzed to determine whether automatic peak detection required manual correction. A complete list of antibodies with their respective dilution ratios is summarized in **Table S4**.

**Cellular thermal shift assay**

HCT116 cells were treated with DMSO or 4 μM TA for 4 h. Cells were resuspended in PBS and divided into six groups, and then heated for 10 min to 55, 57, 59, 61, 63 or 65°C. The samples were lysed by freeze-thaw cycles using liquid nitrogen to obtain cell lysates. After centrifugation at 13,000 g for 10 min, protein content was measured using a BCA kit, and then analyzed by immunoblot with anti-PLK1 antibody. Band intensities were quantified with ImageJ. A complete list of antibodies with their respective dilution ratios is summarized in **Table S4**.

**Drug affinity responsive target stability assay**

HCT116 cells were lysed with IP lysis buffer (Thermo Fisher Scientific, #87788). The lysates were diluted with 10× TNC buffer and treated with TA or DMSO as a control. After incubation for 1 h at room temperature, 5 μg/mL pronase E (Sigma-Aldrich, #P5147) was added into the lysates for a further 15 min at 37°C. Reactions were terminated by adding the 5× loading buffer, and then the samples were evaluated via immunoblot. Band intensities were quantified with ImageJ. A complete list of antibodies with their respective dilution ratios is summarized in **Table S4**.

**PLK1 expression and purification**

The full-length PLK1 and its catalytic domain (residues 13-345) were expressed as N-terminal His₆-MBP fusion proteins using a pET28a-based vector. The polo-box domain (PBD, residues 371-603) was cloned into a pET28a vector with an N-terminal His₆ tag. Site-directed mutants of PLK1 (R57A, R135A, W410A, and F559A) were generated by PCR-based site-directed mutagenesis using the wild-type construct as a template. All constructs were verified by Sanger sequencing. All recombinant proteins were expressed in *E. coli* BL21(AI) cells. Bacteria were cultured in LB medium at 37°C until the OD₆₀₀ reached 0.6-0.8. Protein expression was induced with 0.4 mM IPTG at 16°C for 16 h. The purification procedures for PLK1 were performed as previously described [4, 5]. Cells were harvested by centrifugation, resuspended in lysis buffer (20 mM Tris-HCl, pH 8.0, 500 mM NaCl, 2 mM β-mercaptoethanol), and lysed by sonication. The soluble fractions were clarified by centrifugation at 18,000×g for 40 min at 4°C. Proteins were purified by Ni-NTA affinity chromatography followed by size-exclusion chromatography on a Superdex 200 10/300 GL column equilibrated with gel filtration buffer (20 mM HEPES, pH 8.0, 50 mM NaCl). All recombinant proteins, including the wild-type and mutants, were purified to >95% homogeneity as judged by SDS-PAGE. Protein concentrations were determined by UV absorbance at 280 nm using the calculated extinction coefficients.

**Microscale thermophoresis assay**

MST assays were performed to determine the binding affinities between TA and different forms of PLK1. Purified recombinant proteins (full-length PLK1, PLK1-CD, PLK1-PBD and PLK1 mutants) were labeled using the Monolith™ NT.115 Protein Labeling Kit RED-NHS (NanoTemper Technologies, #MO-L011) according to the manufacturer’s instructions. Labeled proteins were diluted to a final concentration of 200 nM in assay buffer (20 mM HEPES, pH 8.2, 150 mM NaCl). TA was serially diluted in the same assay buffer to generate a 12-point concentration gradient. The diluted TA solutions were then mixed with the labeled PLK1 proteins at a 1:1 ratio and incubated for 30 min at 37°C. Samples were loaded into Monolith standard-treated capillaries, and thermophoresis measurements were carried out at 25°C on a Monolith NT.115 instrument (NanoTemper Technologies) with 20%-50% LED power and medium MST power. Binding curves were recorded in triplicate, and the K_d_ was calculated by fitting the data to a one-site binding model using NT Analysis (NanoTemper Technologies).

**Surface plasmon resonance assay**

The compounds binding to human PLK1 protein were also measured by the SPR using a Biacore 8K instrument (GE Healthcare, Uppsala, Sweden). PLK1 protein (5 mg/mL) dissolved in HEPES buffer (pH 7.4) was immobilized onto a CM5 sensor chip (GE Healthcare), which was pre-activated with a mixture of EDC/NHS for 420 s at a flow rate of 10 μL/min. The reference surface was prepared by replacing PLK1 protein solution with HEPES buffer in the same way. TA was diluted to different concentrations with PBS for binding to PLK1 protein at a flow rate of 20 μL/min for an association phase of 120 s, followed by 300 s dissociation. The dissociation constant K_d_ values were fitted using BIAevaluation.

**Photoaffinity labeling and MS measurements**

Purified PLK1 protein was reconstituted in ultrapure water to a final concentration of 1 mg/mL, followed by incubation with either 10 μM TA-DA or DMSO (control) for 30 min. The sample was subsequently subjected to UV irradiation (365 nm, 10 min) on ice. Probe-labeled protein was then characterized using peptide mapping analysis. After trypsin digestion (overnight, 37 °C), reactions were quenched with trifluoroacetic acid. Fractions were separated on C18 columns and peptides were identified by matrix-assisted laser desorption/ionization (MS and MS/MS analysis).

**Biotinylated pull-down assay**

The synthetic route of TA-Biotin is shown in **Scheme S1**. For the pull-down assay, 2 μg of purified recombinant His-tagged PLK1 protein was incubated with 5 μM biotin, 5 μM free TA, or 5 μM TA-Biotin in 500 μL binding buffer (20 mM Tris-HCl, pH 7.5, 150 mM NaCl, 0.5% NP-40, 1 mM DTT) at 4°C for 4 h with gentle rotation. Subsequently, 30 μL of streptavidin-conjugated magnetic beads were added to each sample and incubated for an additional 2 h at 4°C. The beads were then washed three times with 1 mL binding buffer to remove unbound proteins. Bound proteins were eluted by boiling the beads in 1× SDS sample buffer for 10 min at 95°C. Input and pull-down samples were separated by SDS-PAGE and analyzed by immunoblot using an anti-His antibody. For competitive experiments, recombinant His-PLK1 was pre-incubated with 5 μM free TA or biotin for 1 h prior to the addition of 5 μM TA-Biotin for another 3 h. Bound proteins were detected by immunoblotting with anti-His antibody. A complete list of antibodies with their respective dilution ratios is summarized in **Table S4**.

**Immunofluorescence staining**

Cells were seeded onto 35 mm glass-bottom confocal dishes (NEST) at a density of 8×10^4^ cells per dish and allowed to adhere overnight. After the indicated treatments, cells were washed twice with ice-cold PBS and fixed with 4% paraformaldehyde in PBS for 10 min at room temperature. Following fixation, cells were permeabilized with 0.2% Triton X-100 in PBS for 10 min and blocked with 10% normal goat serum (NGS) in PBS for 1 h at room temperature to minimize non-specific binding. Primary antibodies were diluted in 5% NGS/PBS and incubated with the cells overnight at 4°C. After three washes with PBS, cells were incubated with Alexa Fluor 405-, Alexa Fluor 488- or Alexa Fluor 594-conjugated secondary antibodies (1:500, Abcam) for 1 h at room temperature in the dark. Nuclei were counterstained with DAPI (0.5 μg/mL) for 10 min. Images were acquired using a Zeiss LSM 980 with a 40× immersion objective, collected and processed using Zen Blue. Quantification was performed using ImageJ. A complete list of antibodies with their respective dilution ratios is summarized in **Table S4**.

**Generation of PLK1 or CEP55 stable knockdown cell lines**

To establish stable PLK1- or CEP55-knockdown CRC cell lines, shRNAs specifically targeting human PLK1 or CEP55 and a scrambled negative control (shNC) were cloned into the pLKO.1-puro lentiviral vector. The targeting sequences were as follows: shPLK1: 5’-GTTCTTTACTTCTGGCTATAT-3’; shCEP55: 5’-GCAGCATCAATTGCTTGTAAT-3’. Lentiviral particles were generated by co-transfecting the constructed pLKO.1 plasmids with packaging plasmids (psPAX2 and pMD2.G) into HEK293T cells using Lipofectamine 3000 (Invitrogen). The virus-containing supernatants were harvested at 48 h and 72 h. Cells were transduced with the collected lentiviruses in the presence of 8 μg/mL polybrene (Sigma-Aldrich) to enhance infection efficiency. At 48 h post-infection, stably transduced cells were selected by adding 2 μg/mL puromycin (Sigma-Aldrich) to the culture medium for 7 days. The knockdown efficiency was evaluated via Western blot.

**PLK1 and CEP55 Rescue Experiments**

For rescue experiments, shRNA-resistant PLK1 and CEP55 constructs were generated by introducing synonymous mutations within the corresponding shRNA target sequences without altering the encoded amino acids. The PLK1 target sequence 5′-GTTCTTTACTTCTGGCTATAT-3′ was changed to 5′-GTTTTTCACCTCCGGTTACAT-3′, and the CEP55 target sequence 5′-GCAGCATCAATTGCTTGTAAT-3′ to 5′-GCAACACCAGTTACTCGTCAT-3′. PLK1^KD^ cells were transfected with shRNA-resistant pCMV-HA-PLK1 (human)-Neo, whereas CEP55^KD^ cells were transfected with shRNA-resistant pCMV-CEP55 (human)-3×HA-Neo, pCMV-CEP55^S436A^ (human)-3×HA-Neo, or pCMV-CEP55^S436D^ (human)-3×HA-Neo. The S436A and S436D mutants were generated on the shRNA-resistant CEP55 background. Plasmids were transfected using Lipofectamine 3000 (Invitrogen), and cells were used for subsequent experiments 48 h after transfection.

**PLK1 kinase activity assay**

PLK1 kinase activity was determined using an ADP-Glo™ Kinase Assay (Promega, #V6930). Recombinant PLK1, expressed and purified as described above, was used for the kinase assay. PLK1 (10 ng per well) was incubated with ATP (50 μM) and the indicated peptide substrate (100 μM) in kinase reaction buffer containing 20 mM Tris (pH 8.0), 150 mM NaCl, 20 mM MgCl₂, 0.1 mg/mL BSA, and 5 mM DTT, in a final reaction volume of 10 μL in white 96-well plates. A CEP55-derived peptide encompassing Ser436 (CEP55-WT, PTAALNESLVEAPKA) and the corresponding non-phosphorylatable mutant peptide in which Ser436 was substituted with alanine (CEP55-S436A, PTAALNEALVEAPKA) were synthesized and used as substrates. PLKtide (KKLGEDQAEEISDDLLEDSLSDEDE) was included as a positive-control substrate (SinoBiological, #P41-58). Reactions were carried out at 30°C for 30 min. Control reactions lacking either PLK1 or peptide substrate were included to determine assay background. For inhibition studies, recombinant PLK1 was preincubated with TA (0-40 μM, two-fold serial dilutions) for 15 min at room temperature, after which the kinase reaction was initiated by addition of ATP and peptide substrate. For ATP-dependence experiments, ATP was used at either 50 or 200 μM.

Following the kinase reaction, 10 μL of ADP-Glo reagent was added to each well and incubated at room temperature for 40 min to terminate the kinase reaction and deplete the remaining ATP. Subsequently, 20 μL of Kinase Detection Reagent was added, followed by incubation at room temperature for 60 min. Luminescence was measured using a microplate reader (Promega, GloMax® Explorer GM3500) and expressed as relative luminescence units (RLU). For inhibitor studies, PLK1 kinase activity was normalized to the vehicle-treated control after subtraction of the no-substrate background, with the vehicle-treated group defined as 100% activity. Concentration-response curves were fitted using a four-parameter logistic model to determine IC₅₀ values.

**Evaluation of oxidative stress, mitochondrial dysfunction, lipid peroxidation**

To rigorously investigate the PLK1-dependency of TA-induced cellular stress, a parallel experimental setup was designed using both HCT116 cells and HCT116 PLK1^KD^. Cells were seeded into 6-well or 96-well plates depending on the specific assay requirements and allowed to adhere overnight. Subsequently, both cell lines were exposed to 2 μM TA or an equivalent volume of DMSO. Cells were harvested at different time points post-treatment: 6 h for the evaluation of mitochondrial membrane potential; 12 h for the assessment of intracellular ROS, GSH, ATP, and 8-OHdG levels; and 24 h for the quantification of MDA and 4-HNE. Following these designated treatments, cells or cell lysates were processed for various biochemical evaluations using specific commercial assay kits.

Measurement of intracellular ROS:

Intracellular total ROS levels were specifically quantified utilizing the cell-permeable fluorescent probe DCFH-DA (Beyotime, #S0033S). After TA treatment, the cells were incubated with DCFH-DA diluted in HBSS to a final working concentration of 10 μM. The staining was performed in the dark at 37°C in a humidified incubator for exactly 30 min. Subsequently, the cells were harvested and washed with HBSS to remove unbound extracellular probe. The fluorescence intensity of DCF (indicating intracellular ROS levels) was immediately acquired using an Agilent NovoCyte flow cytometer, and data were analyzed using FlowJo. The fluorescent probe MitoSOX™ (Thermo Fisher Scientific, #M36009) was utilized to evaluate the accumulation of mtROS.

Measurement of mitochondrial membrane potential:

After TA treatment, cells were trypsinized, harvested, and counted. The cell density was adjusted to 1×10⁶ cells/mL. One milliliter of the cell suspension was mixed with TMRE (Beyotime, #C2001S) at a final dilution of 1:1000, and incubated at 37°C for 30 min in the dark. Subsequently, the cells were harvested and washed with HBSS to remove unbound extracellular probe. Cells were immediately analyzed for TMRE fluorescence intensity using an Agilent NovoCyte flow cytometer, and data were analyzed using FlowJo.

Quantification of GSH/GSSG ratio:

Cells were lysed using the specific lysis buffers provided in the respective kits. The ratio of reduced to oxidized glutathione (GSH/GSSG) was determined using a GSSG/GSH Quantification Kit (Dojindo, #G257) according to the manufacturer’s instructions.

Quantification of ATP:

ATP levels were measured using a firefly luciferase-based ATP assay kit (Beyotime, #S0027) according to the manufacturer’s instructions. Luminescence was detected using a Promega luminescence reader. ATP concentrations were calculated based on a standard curve, normalized to the total protein concentration (determined by the BCA protein assay kit), and expressed as nM/mg protein.

Quantification of MDA and 4-HNE:

Briefly, cell homogenates were prepared and clarified by centrifugation. MDA concentration was determined using a Lipid Peroxidation MDA Assay Kit (Beyotime, #S0131S) based on the classic thiobarbituric acid (TBA) reaction method. The absorbance of the resulting chromophore was measured at 532 nm. To ensure accuracy, MDA levels were normalized to the total protein concentration of each lysate (determined by BCA assay) and expressed as μM/mg protein. In parallel, the accumulation of the lipid peroxidation byproduct 4-HNE was assessed using a specific human 4-HNE ELISA kit (ElabScience, #E-EL-0128). The optical density (OD) was recorded at 450 nm using a microplate reader, and absolute 4-HNE concentrations (ng/mL) were calculated from a highly linear standard curve, strictly following the manufacturer’s instructions.

**Assessment of 8-OHdG levels**

HCT116 and HCT116 PLK1^KD^ cells were seeded into 6-well plates and allowed to adhere overnight. Subsequently, both cell lines were exposed to 2 μM TA or an equivalent volume of DMSO. After 12 h of treatment, cell lysates from each group were analyzed using a competitive 8-OHdG ELISA Kit (ElabScience, #E-EL-0028) strictly following the manufacturer’s instructions. The absorbance was recorded at 450 nm using a microplate reader, and the final 8-OHdG concentrations (ng/mL) were calculated based on the standard curve.

**Synergy analysis**

Synergistic effects between TA and L-OHP were assessed using SynergyFinder 3.0. HCT116-OR cells were treated with a 6×6 dose matrix of TA (0-0.2 μM) and L-OHP (0-40 μM) for 24 h. Cell viability was determined by CCK-8 assay. Synergy scores were calculated using the ZIP, Loewe, and Bliss models. Heatmaps of the dose-response matrix and synergy scores were generated, with mean synergy scores and statistical significance (p-values) reported. A synergy score > 10 was considered synergistic.

**Combination index analysis and Fa-CI plot**

The synergistic effect of TA combined with L-OHP was quantitatively evaluated using the Chou-Talalay method. HCT116-OR cells were seeded in 96-well plates and treated for 24 h with a fixed-ratio dose matrix consisting of five concentrations of L-OHP (40, 20, 10, 5, and 2.5 μM) and five concentrations of TA (0.2, 0.1, 0.05, 0.025, and 0.0125 μM). Cell viability was determined using the CCK-8 assay. The fraction affected (Fa) and combination index (CI) values were calculated using CalcuSyn. CI values < 1 indicate synergism, CI = 1 indicates additivity, and CI > 1 indicates antagonism.

**Confocal imaging and quantification of mitochondrial morphology**

1×10^5^ HCT116 and PLK1^KD^ cells were seeded onto glass-bottom confocal dishes and allowed to adhere overnight. The cells were then exposed to either 1 μM TA or an equivalent volume of DMSO for 6 h. Live cells were incubated with the MitoTracker™ Deep Red FM (Beyotime, #C1032-50μg) at a final concentration of 200 nM for 30 min at 37°C in the dark. After staining, the cells were imaged using Zeiss LSM 980. Quantitative morphometric analysis of the mitochondrial networks was performed using ImageJ. The Analyze Particles function was subsequently utilized to extract geometric parameters (area, perimeter, major axis, and minor axis). To rigorously evaluate the degree of mitochondrial fragmentation, two established morphometric indices were calculated: (1) Mitochondrial Aspect Ratio (AR): A measure of mitochondrial length and elongation, calculated as the ratio of the major axis to the minor axis of the fitted ellipse: AR = Major Axis / Minor Axis. (2) Mitochondrial Form Factor (FF): A measure of mitochondrial branching and structural complexity, calculated using the formula: FF = Perimeter² / (4π × Area). For both morphometric parameters, a minimal value of 1 corresponds to a circle, whereas progressively higher values denote highly elongated, continuous, and intricately branched mitochondrial networks.

**Lipid peroxidation and ferrous ion detection**

HCT116 cells (2×10³ cells/well) were seeded in 24-well plates with cell slides in a total volume of 0.5 mL of medium for 24 h. Serially diluted compounds were added to the cells. After treatment with compounds for 24 h, the cell slides were washed three times with HBSS. Then, 200 μL of 1 μM Liperfluo (#L248, Dojindo) or FerroOrange (#F374, Dojindo) solution diluted with serum-free medium was added to each well and incubated at 37°C in a humidified atmosphere with 5% CO₂ for 30 min. DAPI (Beyotime, #C1002) was used to visualize the nuclei. Image acquisition was achieved using a NIKON ECLIPSE Ti2 confocal fluorescence microscope (Nikon, Japan).

**Quantitative real-time PCR**

Total mRNA was extracted using TRIzol (Sigma-Aldrich, #T9424) and reverse-transcribed with PrimeScriptTM RT reagent Kit (TaKaRa, #RR037A). RT-PCR analyses were performed using TB Green Premix Ex TaqTM II (TaKaRa, #RR820A). GAPDH was used for the normalization of the qPCR data. Data were analyzed with the 2^-ΔΔCt^ method. Primers were listed in **Table S5**.

**Cell-cycle analysis**

Cell-cycle distribution was analyzed by EdU/DAPI staining followed by flow cytometry (EdU, Elabscience, #E-CK-A370; DAPI, Beyotime, # C1002). 2×10^4^ HCT116-OR cells were seeded in 6-well plates and allowed to adhere overnight. Cells were then treated with DMSO, 0.2 μM TA, or the combination of 0.2 μM TA and 10 μM L-OHP for 24 h. Two hours before cell harvesting, EdU was added to the culture medium at a final concentration of 10 μM and incubated with the cells for 2 h. Cells were then collected, fixed, and permeabilized, followed by click-reaction labeling of incorporated EdU according to the manufacturer’s instructions. Cellular DNA was subsequently stained with 5 μg/mL DAPI for 10 min at room temperature in the dark. Samples were analyzed by flow cytometry, and the percentages of cells in G0/G1, S, and G2/M phases were determined based on EdU incorporation and DNA content.

**Apoptosis analysis**

Apoptosis was assessed by Annexin V-FITC/PI double staining followed by flow cytometry. 2×10^4^ HCT116-OR cells were seeded in 6-well plates and treated with vehicle, 0.2 μM TA, or the combination of 0.2 μM TA and 10 μM L-OHP for 24 h. Both floating and adherent cells were collected, washed twice with cold PBS, and resuspended in binding buffer. Cells were then incubated with Annexin V-FITC and propidium iodide (PI) according to the manufacturer’s instructions in the dark at room temperature (Beyotime, # C1062S). Samples were analyzed by flow cytometry within 20 min after staining. Annexin V⁻/PI⁻ cells were defined as viable cells, Annexin V⁺/PI⁻ cells as early apoptotic cells, and Annexin V⁺/PI⁺ cells as late apoptotic or secondary necrotic cells. Total apoptotic cells were calculated as the sum of early and late apoptotic populations.

**Clinical data acquisition, integration, and batch effect correction**

To comprehensively evaluate the clinical relevance of PLK1 in CRC chemoresistance, transcriptomic data and corresponding clinical annotations were systematically retrieved from the GEO database. Seven independent CRC patient cohorts (GSE104645, GSE19860, GSE39582, GSE81653, GSE106584, GSE28702, and GSE72970), comprising a total of 408 samples, were included in this study. Prior to data integration, raw microarray data were processed using platform-specific normalization procedures. For datasets generated on Affymetrix platforms (GSE19860, GSE28702, GSE39582, GSE72970, GSE81653, and GSE106584), raw CEL files were processed using the Robust Multi-array Average (RMA) algorithm implemented in the *affy* or *oligo* R package, as appropriate for the corresponding platform, including background correction, quantile normalization, and Log2 transformation. For the Agilent microarray dataset (GSE104645), raw signal intensities were background-corrected using the normexp method, quantile-normalized between arrays, and Log2-transformed using the *limma* R package.

Following preprocessing, the normalized Log2 expression matrices were merged based on common gene symbols. To minimize non-biological technical variation across different microarray platforms and cohorts, batch effects were corrected using the empirical Bayes ComBat algorithm implemented in the *sva* R package. To preserve biological variation associated with oxaliplatin response, treatment response status (Responder vs. Non-Responder) was included as a biological covariate in the ComBat model. The effectiveness of batch-effect correction was assessed using boxplots, density plots, and principal component analysis (PCA) before and after correction.

**Patient stratification, extreme phenotype enrichment, and OPLS-DA**

Patients from the integrated cohort were stratified into “Responder” and “Non-Responder” groups based on Response Evaluation Criteria in Solid Tumors (RECIST) and progression-free survival (PFS) records. The predictive efficacy of PLK1 for chemoresistance was assessed by plotting the Receiver Operating Characteristic (ROC) curve and calculating the Area Under the Curve (AUC). To further discern the specific molecular signatures driving resistance while minimizing the profound intrinsic heterogeneity of clinical tumors, an “extreme phenotype enrichment” strategy was implemented. Briefly, we isolated a core cohort (n = 245) comprising the top 60% PLK1-high patients within the Non-Responder group and the top 60% PLK1-low patients within the Responder group. Orthogonal Partial Least Squares Discriminant Analysis (OPLS-DA) was subsequently performed using the *ropls* R package on both the entire cohort and the refined core cohort to evaluate the segregation of transcriptomic profiles between sensitive and resistant phenotypes using the top 2000 features by median absolute deviation (MAD). Model quality was assessed using R^2^Y (goodness of fit) and Q^2^ (predictive ability) parameters, alongside permutation testing.

**Pathway enrichment and network visualization**

Differentially expressed genes (DEGs) identified from the core cohort were subjected to integrative pathway enrichment analysis, incorporating GO-CC, KEGG, and Reactome databases. To elucidate the sequential biological trajectory of PLK1-driven resistance, the significantly enriched pathways were functionally categorized into “Damage & Stress”, “Membrane remodeling & Vesicle budding”, and “Clearance” modules. The complex regulatory network connecting the specific DEGs to these enriched functional modules was visualized using a customized bubble-Sankey diagram generated via the *ggplot2* R package.

**Subcellular fractionation and isolation of micronuclei and MDVs**

To quantify the extrusion of damaged DNA and mitochondrial components, subcellular fractionation was performed utilizing a rigorously optimized differential centrifugation and ultracentrifugation protocol. Briefly, approximately 5×10^7^ cells from each treatment group were harvested, washed twice with ice-cold PBS, and resuspended in a mild hypotonic lysis buffer (10 mM Tris-HCl pH 7.5, 10 mM NaCl, 3 mM MgCl_2_, and 0.1% NP-40) to selectively rupture the plasma membrane while preserving the integrity of the nuclear envelope and organelle membranes. After incubation on ice for 10 min, the cell lysates were subjected to sequential centrifugation steps at 4°C. Isolation of primary nuclei: The homogenate was centrifuged at 800×g for 10 min. The resulting pellet, containing intact primary nuclei and unbroken cells, was collected. Isolation of micronuclei: The supernatant was transferred to a new pre-chilled tube and centrifuged at 10,000×g for 15 min. The resulting pellet, heavily enriched with heavy membrane fractions, mitochondria, and micronuclei, was collected and designated as the micronuclear fraction for Alu DNA quantification. Isolation of MDVs: To isolate the nanoscale MDVs, the post-mitochondrial supernatant from the previous step was carefully collected and subjected to ultracentrifugation at 100,000×g for 70 min at 4°C using a Thermo Scientific™ Sorvall™ MTX 150. The resulting vesicle-enriched pellet was resuspended in a minimal volume of PBS and designated as the MDV fraction for subsequent mtDNA quantification. Total DNA from the isolated subcellular fractions was extracted using a commercial Genomic DNA Extraction Kit (Thermo Fisher Scientific, #A66428) following the manufacturer’s instructions. The concentration and purity of the extracted DNA were assessed using a NanoDrop spectrophotometer (Thermo Fisher Scientific). qPCR was performed to detect the relative abundance of nuclear-derived Alu sequences and mtDNA. To accurately quantify the degree of vesicular extrusion and account for variations in initial cell numbers, the abundance of target DNA in the respective subcellular fractions was normalized to the total corresponding DNA levels amplified from the WCL of the same sample, utilizing the 2^-ΔΔCt^ method. Primers were listed in **Table S5**.

**Transmission electron microscopy**

Tumor tissues were collected immediately after euthanasia and cut into small pieces (approximately 1 mm³). The samples were fixed with 2.5% glutaraldehyde in 0.1 M phosphate buffer (pH 7.4) for 2 h at 4°C. After washing three times with phosphate buffer, the tissues were post-fixed with 1% osmium tetroxide for 2 h at room temperature. Samples were then dehydrated in a graded series of ethanol (30%, 50%, 70%, 80%, 90%, 95%, and 100%), followed by infiltration and embedding in Epon 812 resin. Ultrathin sections (60-70 nm) were cut using an ultramicrotome (Leica EM UC7), mounted on copper grids, and stained with 2% uranyl acetate for 15 min and 0.3% lead citrate for 10 min. Sections were examined and imaged using a FEI Tecnai G2 20 Twin transmission electron microscope operating at 200 kV.

**Statistical analysis**

All quantitative data are presented as the mean ± s.d. or s.e.m. (specified in figure legends) from at least three independent biological replicates. Statistical analyses and graphical representations were performed using GraphPad Prism 9.0 and R 4.5.2. The normal distribution of the data and homogeneity of variances were appropriately checked prior to the analyses. For comparisons between two independent experimental groups, statistical significance was determined using a two-tailed unpaired Student’s t-test. If the clinical or transcriptomic data did not follow a normal distribution, the Mann-Whitney U test was applied as a non-parametric alternative. For comparisons involving three or more groups with a single independent variable, statistical analysis was performed using one-way ANOVA followed by Tukey’s multiple comparisons test. For complex experimental designs involving two independent variables, statistical significance was determined by two-way ANOVA followed by Šídák’s or Tukey’s multiple comparisons test, depending on the specific comparison requirements.

For high-throughput proteomic and transcriptomic datasets, multiple-testing correction was applied to control the FDR. In differential expression analyses, p-values were adjusted using the Benjamini–Hochberg (BH) method, with an FDR-adjusted p-value < 0.05 considered statistically significant. For pathway enrichment analyses, including GO, KEGG, and Reactome, p-values were corrected for multiple testing using the BH method, and an adjusted p-value (p.adjust) < 0.05 was used as the significance threshold. Multiple comparisons in the Mantel tests used for pathway–clinical correlation analyses were likewise corrected using the BH method. For bioinformatics and clinical datasets, correlation analyses were performed using Pearson’s correlation for normally distributed variables and Spearman’s correlation for non-normally distributed variables. Kaplan-Meier survival analysis, coupled with the log-rank test, was conducted to compare overall survival (OS) across subgroups stratified by optimal PLK1 expression cut-offs. A p-value < 0.05 was considered statistically significant for conventional statistical analyses, whereas an FDR-adjusted p-value < 0.05 was used for high-throughput omics and pathway enrichment analyses. “ns” indicates no significant difference.

**Chemistry experimental procedures and data**

All reagents were procured from commercial sources and used without further purification. Silica gel GF254 and silica gel (200-300 mesh) were respectively used for thin-layer chromatography (TLC) and column chromatography. NMR spectra were recorded on a Bruker AM-400 spectrometer (CDCl_3_ = 7.26 ppm for ^1^H NMR and 77.16 ppm for ^13^C NMR, Pyridine-*d_5_* = 7.16 ppm for ^1^H NMR and 128.06 ppm for ^13^C NMR). TA was isolated from *Tubocapsicum anomalum*. 4-Pentynoic acid, 6-Heptynoic acid, Biotin, 4-Bromo-1,8-naphthalic anhydride, Dimethylamine (40 wt.% solution in water), 3-(3-Methyl-3*H*-diazirin-3-yl) propanoic acid, 1-(3-Dimethylaminopropyl)-3-ethylcarbodiimide hydrochloride (EDCI), 4-Dimethylaminopyridine (DMAP), CuSO_4_, *L*-Ascorbic acid sodium, *N*, *N*-Diisopropylethylamine (DIPEA) were purchased from Energy-chemical. Azide-nPEG-amines (n = 2/3) were purchased from Tokyo Chemical Industry. The synthesis of N_3_-Linker-E3-Ligands refers to the relevant content in the research article about PD-L1 degradation agents [6].

Representative synthetic procedures for **TA-PROTAC** probes were illustrated in **Figure 2A**. Detailed reaction condition information and characterization data were described as follows:

**Synthesis of (4*S*, 4a*S*, 5a*R*, 6a*R*, 8*R*, 9*S*, 11a*S*, 11b*R*)-9-((*S*)-1-((*R*)-4, 5-dimethyl-6-oxo-3, 6-dihydro-2*H*-pyran-2-yl) ethyl)-8-hydroxy-9, 11b-dimethyl-1-oxo-1, 4, 5a, 6, 6a, 7, 8, 9, 10, 11, 11a, 11b-dodecahydrocyclopenta [1, 2] phenanthrol [8a, 9-*b*] oxiren-4-yl hept-6-ynoate** (**TA-ester**). TA (100.0 mg, 0.21 mmol), 6-Heptynoic acid (31.5 mg, 0.25 mmol), EDCI (80.5 mg, 0.42 mmol), and DMAP (5.0 mg, 0.04 mmol) were added to 5 mL of Dichloromethane, and the reaction continued overnight at room temperature. Added 25 mL of Dichloromethane to the reaction system. The organic phase was washed with H_2_O (10 mL*2), dried over anhydrous Na_2_SO_4_, and concentrated in a vacuum to give a residue. The residue was purified by silica gel chromatography (Dichloromethane : Methanol = 60 : 1, v/v) to yield the desired compound, a white solid with a yield of 66%. ^1^H NMR (600 MHz, CDCl_3_) *δ* 7.08 (dd, *J* = 9.8, 6.1 Hz, 1H), 6.26 (d, *J* = 9.8 Hz, 1H), 4.69 (d, *J* = 6.1 Hz, 1H), 4.37 (m, 1H), 4.13 (t, *J* = 7.7 Hz, 1H), 3.29 (d, *J* = 2.0 Hz, 1H), 2.49 – 2.41 (m, 2H), 2.40 – 2.33 (m, 4H), 2.19 (m, 3H), 2.14 (td, *J* = 13.4, 12.7, 6.1 Hz, 3H), 2.07 – 2.00 (m, 1H), 1.95 (t, *J* = 2.6 Hz, 1H), 1.91 (s, 3H), 1.86 (d, *J* = 2.2 Hz, 3H), 1.75 – 1.70 (m, 2H), 1.68 (d, *J* = 3.1 Hz, 1H), 1.56 – 1.51 (m, 2H), 1.35 (s, 3H), 1.31 – 1.26 (m, 1H), 1.17 (dd, *J* = 12.4, 4.7 Hz, 1H), 1.12 (s, 3H), 1.09 – 1.05 (m, 1H), 0.97 (d, *J* = 7.4 Hz, 3H).

**Synthesis of (4*S*, 4a*S*, 5a*R*, 6a*R*, 8*R*, 9*S*, 11a*S*, 11b*R*)-9-((*S*)-1-((*R*)-4, 5-dimethyl-6-oxo-3, 6-dihydro-2*H*-pyran-2-yl)ethyl)-8-hydroxy-9, 11b-dimethyl-1-oxo-1, 4, 5a, 6, 6a, 7, 8, 9, 10, 11, 11a, 11b-dodecahydrocyclopenta [1, 2] phenanthro [8a, 9-*b*] oxiren-4-yl 5-(1-(2-(2-(2-(2-((2-(2, 6-dioxopiperidin-3-yl)-1, 3-dioxoisoindolin-4-yl) amino) ethoxy) ethoxy) ethoxy) ethyl)-1*H*-1, 2, 3-triazol-4-yl) pentanoate** (**TA-P**). TA ester (57.7 mg, 0.10 mmol) and N_3_-3P-Pomalidomide (57.0 mg, 0.12 mmol) were dissolved in THF (1 mL), followed by the addition of water (1 mL), CuSO_4_ (20.0 mg, 0.13 mmol), and *L*-Ascorbic acid sodium (59.4 mg, 0.30 mmol) to the reaction system. TLC detection showed that the reaction is complete, THF was removed under reduced pressure, 10 mL of Dichloromethane was added to the residue, the organic phase was washed with H_2_O (3 mL*2), collected the organic phase, and dried with anhydrous Na_2_SO_4_. The crude product was purified by silica gel chromatography (Dichloromethane: Methanol = 20 : 1, v/v) to obtain a yellow solid product, with a yield of 53%.

^1^H NMR (600 MHz, CDCl_3_) *δ* 8.36 (s, 1H), 7.55 (d, *J* = 9.8 Hz, 1H), 7.50 (dd, *J* = 8.5, 7.1 Hz, 1H), 7.11 (d, *J* = 7.1 Hz, 1H), 7.07 (dd, *J* = 9.8, 6.1 Hz, 1H), 6.92 (d, *J* = 8.5 Hz, 1H), 6.26 (d, *J* = 9.8 Hz, 1H), 4.95 – 4.89 (m, 1H), 4.68 (d, *J* = 6.1 Hz, 1H), 4.51 (d, *J* = 5.0 Hz, 2H), 4.34 (s, 1H), 4.13 (t, *J* = 7.6 Hz, 1H), 3.87 (t, *J* = 4.7 Hz, 2H), 3.72 (t, *J* = 5.3 Hz, 2H), 3.67 – 3.59 (m, 9H), 3.47 (t, *J* = 5.4 Hz, 2H), 3.28 (s, 1H), 3.10 (dd, *J* = 7.3, 4.8 Hz, 1H), 2.89 (d, *J* = 14.2 Hz, 1H), 2.80 – 2.71 (m, 4H), 2.48 – 2.38 (m, 3H), 2.35 (m, 3H), 2.20 (m, 1H), 2.15 – 2.10 (m, 3H), 2.03 (m, 1H), 1.92 (m, 3H), 1.86 (d, *J* = 2.4 Hz, 3H), 1.66 (d, *J* = 12.6 Hz, 4H), 1.33 (s, 3H), 1.31 – 1.25 (m, 2H), 1.22 (d, *J* = 7.9 Hz, 1H), 1.12 (s, 3H), 1.10 – 1.04 (m, 1H), 0.98 (dd, *J* = 13.8, 6.7 Hz, 3H). ^13^C NMR (101 MHz, CDCl_3_) *δ* 201.45, 178.28, 172.12, 171.08, 169.28, 168.40, 167.39, 165.95, 149.50, 139.72, 138.60, 136.08, 134.60, 133.54, 131.12, 122.53, 121.68, 117.28, 112.55, 110.32, 82.22, 78.67, 71.88, 70.76, 70.64, 70.57, 69.53, 61.27, 60.53, 53.33, 50.85, 50.19, 48.89, 47.97, 45.83, 43.54, 42.41, 40.92, 40.62, 34.37, 33.72, 31.43, 30.38, 29.40, 28.60, 25.37, 25.17, 24.31, 24.06, 22.96, 22.79, 20.55, 14.96, 12.50, 12.36, 8.62.

**Synthesis of (4*S*, 4a*S*, 5a*R*, 6a*R*, 8*R*, 9*S*, 11a*S*, 11b*R*)-9-((*S*)-1-((*R*)-4, 5-dimethyl-6-oxo-3, 6-dihydro-2*H*-pyran-2-yl) ethyl)-8-hydroxy-9, 11b-dimethyl-1-oxo-1, 4, 5a, 6, 6a, 7, 8, 9, 10, 11, 11a, 11b-dodecahydrocyclopenta [1, 2] phenanthro [8a, 9-*b*] oxiren-4-yl 5-(1-(4-(((*S*)-1-((2*S*, 4*R*)-4-hydroxy-2-(((*R*)-1-(4-(4-methylthiazol-5-yl) phenyl) ethyl) carbamoyl) pyrrolidin-1-yl)-3, 3-dimethyl-1-oxobutan-2-yl) amino)-4-oxobutyl)-1*H*-1, 2, 3-triazol-4-yl) pentanoate** (**TA-V**). The reaction feeding and work-up steps refer to the synthesis process of **TA-P**. **TA-V** is a light yellow solid with a yield of 58%. ^1^H NMR (600 MHz, CDCl_3_) *δ* 8.71 (s, 1H), 7.39 – 7.35 (m, 4H), 7.07 (dd, *J* = 9.8, 6.1 Hz, 1H), 6.61 (s, 1H), 6.26 (d, *J* = 9.7 Hz, 1H), 5.09 (t, *J* = 6.9 Hz, 1H), 4.77 (s, 1H), 4.68 (d, *J* = 6.0 Hz, 1H), 4.51 (s, 1H), 4.41 (s, 1H), 4.37 (m, 3H), 4.13 (t, *J* = 7.6 Hz, 1H), 4.07 (s, 1H), 3.53 (d, *J* = 11.4 Hz, 1H), 3.28 (s, 1H), 2.71 (s, 2H), 2.60 – 2.55 (m, 1H), 2.52 (s, 3H), 2.45 (s, 2H), 2.40 – 2.31 (m, 5H), 2.25 (d, *J* = 10.3 Hz, 1H), 2.16 (m, 10H), 2.04 (m, 2H), 1.91 (s, 3H), 1.85 (d, *J* = 2.7 Hz, 3H), 1.67 (s, 4H), 1.52 (d, *J* = 6.9 Hz, 3H), 1.33 (s, 3H), 1.30 – 1.28 (m, 1H), 1.27 – 1.25 (m, 1H), 1.12 (s, 3H), 1.06 (d, *J* = 7.3 Hz, 2H), 0.97 (d, *J* = 7.4 Hz, 3H), 0.86 (s, 9H). ^13^C NMR (101 MHz, CDCl_3_) *δ* 201.25, 172.53, 172.07, 169.61, 166.31, 149.78, 143.09, 140.28, 138.98, 134.58, 133.79, 129.54, 126.74, 121.69, 82.19, 78.68, 77.23, 71.94, 70.06, 61.26, 60.54, 58.33, 58.14, 56.65, 53.29, 49.84, 47.98, 47.18, 43.54, 42.52, 40.90, 40.63, 35.73, 34.73, 34.31, 33.76, 32.49, 32.00, 31.83, 30.38, 29.68, 29.41, 29.22, 28.51, 26.59, 26.40, 26.04, 25.35, 25.21, 24.33, 24.06, 22.95, 21.97, 20.56, 15.92, 14.97, 12.47, 12.36.

**Synthesis of (4*S*, 4a*S*, 5a*R*, 6a*R*, 8*R*, 9*S*, 11a*S*, 11b*R*)-9-((*S*)-1-((*R*)-4, 5-dimethyl-6-oxo-3, 6-dihydro-2*H*-pyran-2-yl) ethyl)-8-hydroxy-9, 11b-dimethyl-1-oxo-1, 4, 5a, 6, 6a, 7, 8, 9, 10, 11, 11a, 11b-dodecahydrocyclopenta [1, 2] phenanthro [8a, 9-*b*] oxiren-4-yl 5-(1-(1-(4-((4*R*, 5*S*)-4, 5-bis(4-chlorophenyl)-2-(2-isopropoxy-4-methoxyphenyl)-4, 5-dihydro-1*H*-imidazole-1-carbonyl)-2-oxopiperazin-1-yl)-2-oxo-6, 9, 12-trioxa-3-azatetradecan-14-yl)-1*H*-1, 2, 3-triazol-4-yl) pentanoate** (**TA-N**). The reaction feeding and work-up steps refer to the synthesis process of **TA-P**. **TA-N** is a light yellow solid with a yield of 38%. ^1^H NMR (600 MHz, CDCl_3_) *δ* 7.71 – 7.51 (m, 1H), 7.09 (m, 5H), 6.87 (s, 3H), 6.64 (m, 1H), 6.49 (s, 1H), 6.25 (dd, *J* = 9.5, 4.1 Hz, 1H), 5.69 (s, 1H), 4.68 (d, *J* = 5.8 Hz, 2H), 4.37 (m, 1H), 4.12 (s, 1H), 3.89 (m, 7H), 3.63 – 3.48 (m, 12H), 3.38 (m, 2H), 3.29 (s, 1H), 3.18 (s, 1H), 2.83 – 2.52 (m, 2H), 2.49 – 2.29 (m, 7H), 2.21 – 2.17 (m, 1H), 2.15 – 2.10 (m, 2H), 2.03 (d, *J* = 17.4 Hz, 2H), 1.91 (s, 3H), 1.85 (s, 3H), 1.69 (s, 4H), 1.42 (d, *J* = 8.4 Hz, 3H), 1.37 – 1.32 (m, 6H), 1.29 (d, *J* = 1.5 Hz, 2H), 1.26 (d, *J* = 6.9 Hz, 2H), 1.23 (d, *J* = 8.7 Hz, 1H), 1.16 (dd, *J* = 12.3, 4.4 Hz, 1H), 1.12 (s, 3H), 1.08 (d, *J* = 10.1 Hz, 1H), 0.96 (dd, *J* = 7.4, 1.9 Hz, 3H). ^13^C NMR (101 MHz, CDCl_3_) *δ* 201.23, 172.51, 167.69, 166.33, 162.93, 153.71, 151.48, 149.79, 148.43, 144.21, 140.39, 139.02, 138.24, 134.59, 133.82, 133.11, 128.97, 128.12, 124.40, 123.86, 121.69, 119.07, 116.39, 105.12, 98.23, 82.20, 80.20, 78.67, 75.93, 75.04, 71.95, 71.22, 70.51, 70.29, 69.63, 68.58, 66.67, 66.05, 63.92, 61.28, 60.58, 57.32, 55.89, 53.29, 50.09, 48.00, 46.78, 43.55, 42.69, 42.42, 40.98, 40.67, 39.28, 36.75, 34.26, 33.84, 33.00, 31.91, 31.43, 30.40, 30.19, 29.69, 29.31, 27.26, 25.39, 24.43, 24.07, 23.42, 22.96, 22.61, 20.59, 15.06, 13.92, 12.44, 12.38.

**Synthesis of methyl (3-(11-(4-(5-(((4*S*, 4a*S*, 5a*R*, 6a*R*, 8*R*, 9*S*, 11a*S*, 11b*R*)-9-((*S*)-1-((*R*)-4, 5-dimethyl-6-oxo-3, 6-dihydro-2*H*-pyran-2-yl) ethyl)-8-hydroxy-9,11b-dimethyl-1-oxo-1, 4, 5a, 6, 6a, 7, 8, 9, 10, 11, 11a, 11b-dodecahydrocyclopenta [1, 2] phenanthro [8a, 9-*b*] oxiren-4-yl) oxy)-5-oxopentyl)-1*H*-1, 2, 3-triazol-1-yl) undecanamido)-2-hydroxy-4-phenylbutanoyl) leucinate** (**TA-B**). The reaction feeding and work-up steps refer to the synthesis process of **TA-P**. **TA-B** is a light yellow solid with a yield of 54%. ^1^H NMR (600 MHz, CDCl_3_) *δ* 7.33 (d, *J* = 8.7 Hz, 2H), 7.28 (d, *J* = 7.8 Hz, 1H), 7.26 (s, 1H), 7.21 (m, 4H), 7.08 (dd, *J* = 9.8, 6.1 Hz, 1H), 6.25 (dd, *J* = 9.6, 3.8 Hz, 2H), 4.68 (d, *J* = 6.1 Hz, 1H), 4.60 – 4.54 (m, 1H), 4.37 (m, 1H), 4.32 (s, 2H), 4.20 – 4.16 (m, 2H), 4.13 (t, *J* = 7.6 Hz, 1H), 3.72 (s, 3H), 3.29 (d, *J* = 1.9 Hz, 1H), 3.08 (t, *J* = 8.4 Hz, 2H), 2.77 – 2.68 (m, 2H), 2.45 – 2.32 (m, 6H), 2.19 (dd, *J* = 17.7, 3.4 Hz, 1H), 2.17 – 2.09 (m, 3H), 2.05 (td, *J* = 7.7, 5.6 Hz, 2H), 1.91 (s, 3H), 1.86 – 1.85 (m, 3H), 1.72 – 1.62 (m, 6H), 1.61 – 1.56 (m, 2H), 1.46 (t, *J* = 7.4 Hz, 2H), 1.34 (s, 3H), 1.32 – 1.28 (m, 6H), 1.25 – 1.17 (m, 8H), 1.12 (s, 3H), 0.97 (d, *J* = 7.4 Hz, 3H), 0.92 (d, *J* = 6.2 Hz, 3H), 0.90 (d, *J* = 6.2 Hz, 3H). ^13^C NMR (101 MHz, CDCl_3_) *δ* 201.22, 175.53, 172.76, 172.52, 166.36, 149.78, 140.30, 139.02, 138.01, 134.56, 133.77, 129.19 (2), 128.54 (2), 126.67, 121.70, 82.20, 78.68, 77.23, 73.82, 71.91, 69.74, 68.07, 64.21, 61.26, 60.52, 56.09, 53.28, 52.31, 50.40, 47.97, 43.55, 42.78, 42.67, 41.19, 40.90, 40.64, 39.18, 36.38, 35.55, 34.27, 33.79, 30.39, 30.18, 29.24, 29.18, 29.14, 29.06, 28.90, 26.53, 25.49, 25.32, 24.79, 24.37, 24.05, 22.95, 22.88, 21.65, 20.56, 14.96, 12.44, 12.36.

Representative synthetic procedures for the **TA-Biotin** probe were illustrated in **Supplementary Scheme 1**. Detailed reaction condition information and characterization data were described as follows:

**Synthesis of** **(4*S*, 4a*S*, 5a*R*, 6a*R*, 8*R*, 9*S*, 11a*S*, 11b*R*)-9-((*S*)-1-((*R*)-4, 5-dimethyl-6-oxo-3, 6-dihydro-2*H*-pyran-2-yl)ethyl)-8-hydroxy-9, 11b-dimethyl-1-oxo-1, 4, 5a, 6, 6a, 7, 8, 9, 10, 11, 11a, 11b-dodecahydrocyclopenta [1, 2] phenanthrol [8a, 9-*b*] oxiren-4-yl pent-4-ynoate** (**TA-yne**). The reaction feeding and work-up steps refer to the synthesis process of the **TA-ester**. The difference is using 4-Pentynoic acid instead of 6-Heptynoic acid during the feeding process. **TA-yne** was a white solid with a yield of 62%. ^1^H NMR (600 MHz, CDCl_3_) *δ* 7.08 (dd, *J* = 9.8, 6.1 Hz, 1H), 6.27 (d, *J* = 9.8 Hz, 1H), 4.71 (d, *J* = 6.1 Hz, 1H), 4.39 (m, 1H), 4.13 (dd, *J* = 8.3, 7.2 Hz, 1H), 3.30 (d, *J* = 1.9 Hz, 1H), 2.48 (d, *J* = 2.6 Hz, 1H), 2.47 (d, *J* = 2.6 Hz, 1H), 2.46 – 2.44 (m, 1H), 2.44 – 2.41 (m, 1H), 2.39 – 2.33 (m, 2H), 2.22 – 2.18 (m, 1H), 2.17 – 2.15 (m, 1H), 2.15 – 2.11 (m, 2H), 2.07 – 2.01 (m, 1H), 1.98 (t, *J* = 2.6 Hz, 1H), 1.91 (d, *J* = 1.5 Hz, 3H), 1.87 – 1.84 (m, 3H), 1.68 (m, 1H), 1.35 (s, 3H), 1.32 – 1.28 (m, 1H), 1.28 – 1.24 (m, 1H), 1.19 – 1.15 (m, 1H), 1.12 (s, 3H), 1.10 – 1.05 (m, 1H), 0.97 (d, *J* = 7.4 Hz, 3H).

**Synthesis of *N*-(2-(2-(2-azidoethoxy) ethoxy) ethyl)-5-((3a*S*, 4*S*, 6a*R*)-2-oxohexahydro-1*H*-thieno [3, 4-*d*] imidazol-4-yl) pentanamide** (**N_3_-2P-Biotin**). Biotin (244.3 mg, 1.0 mmol) was added to a round-bottomed flask and dissolved in 10 mL of DMSO. EDCI (383.4 mg, 2.0 mmol), HOBt (270.3 mg, 2.0 mmol), Azide-2PEG-amine (261.3 mg, 1.5 mmol), and DIPEA (697 μL, 4.0 mmol) were sequentially added to the reaction vessel and heated to 45°C for overnight. The reaction mixture was poured into H_2_O (30 mL), then the mixture was extracted with Ethyl acetate (10 mL*3). The organic phase was washed with brine (10 mL*2), dried over anhydrous Na_2_SO_4_, and concentrated in a vacuum to give a residue. The residue was purified by silica gel chromatography (Dichloromethane : Methanol = 20 : 1, v/v) to yield the desired compound, a white solid with a yield of 34%. ^1^H NMR (600 MHz, CDCl_3_) *δ* 6.64 (s, 1H), 6.51 (s, 1H), 4.54 – 4.49 (m, 1H), 4.33 (dd, *J* = 7.7, 4.5 Hz, 1H), 3.69 (d, *J* = 4.9 Hz, 2H), 3.67 – 3.63 (m, 4H), 3.58 (t, *J* = 5.1 Hz, 2H), 3.48 (s, 4H), 3.47 – 3.43 (m, 2H), 3.42 – 3.39 (m, 2H), 3.15 (td, *J* = 7.4, 4.4 Hz, 1H), 2.91 (dd, *J* = 12.8, 4.8 Hz, 1H), 2.25 – 2.21 (m, 2H), 1.71 – 1.66 (m, 2H), 1.45 (m, 2H).

**Synthesis of (4*S*, 4a*S*, 5a*R*, 6a*R*, 8*R*, 9*S*, 11a*S*, 11b*R*)-9-((*S*)-1-((*R*)-4, 5-dimethyl-6-oxo-3, 6-dihydro-2*H*-pyran-2-yl)ethyl)-8-hydroxy-9, 11b-dimethyl-1-oxo-1, 4, 5a, 6, 6a, 7, 8, 9, 10, 11, 11a, 11b-dodecahydrocyclopenta [1, 2] phenanthrol [8a, 9-*b*] oxiren-4-yl 3-(1-(2-(2-(2-(5-((3a*S*, 4*S*, 6a*R*)-2-oxohexahydro-1*H*-thieno [3, 4-d] imidazol-4-yl) pentanamido) ethoxy) ethoxy) ethyl)-1*H*-1, 2, 3-triazol-4-yl) propanoate** (**TA-Biotin**). The TA-yne and N_3_-2P-Biotin were used as raw materials to obtain the target compound by Click Chemistry reaction. The reaction feeding and work-up steps refer to the synthesis process of the **TA-P**. **TA-Biotin** was a white solid with a yield of 68%. ^1^H NMR (600 MHz, CDCl_3_) *δ* 7.07 (s, 1H), 6.68 (s, 1H), 6.27 (d, *J* = 9.2 Hz, 1H), 4.73 (s, 1H), 4.61 (s, 2H), 4.42 – 4.36 (m, 1H), 4.13 (t, *J* = 7.7 Hz, 1H), 4.02 – 3.91 (m, 2H), 3.59 (d, *J* = 17.3 Hz, 4H), 3.53 (s, 2H), 3.43 (s, 2H), 3.32 (d, *J* = 14.0 Hz, 2H), 2.80 (s, 6H), 2.44 (m, 2H), 2.36 (t, *J* = 12.8 Hz, 2H), 2.28 (d, *J* = 16.9 Hz, 2H), 2.21 – 2.09 (m, 4H), 2.08 – 2.01 (m, 1H), 1.91 (s, 3H), 1.85 (s, 3H), 1.74 – 1.63 (m, 4H), 1.51 – 1.42 (m, 2H), 1.32 (s, 3H), 1.26 (s, 6H), 1.17 – 1.14 (m, 3H), 1.12 (s, 3H), 1.07 (t, *J* = 11.3 Hz, 1H), 0.97 (d, *J* = 7.3 Hz, 3H). ^13^C NMR (101 MHz, CDCl_3_) *δ* 201.17, 171.65, 166.43, 150.51, 140.05, 139.09, 134.50, 133.94, 121.65, 81.55, 78.68, 72.91, 70.95, 70.15, 69.96, 68.84, 68.30, 61.66, 60.63, 53.22, 47.94, 47.10, 43.50, 41.71, 40.87, 40.68, 39.20, 36.01, 35.36, 34.16, 33.04, 32.93, 30.97, 30.36, 30.18, 29.69, 29.35, 28.27, 27.14, 27.03, 25.63, 25.30, 24.04, 23.33, 22.69, 20.61, 15.85, 14.12, 11.30.

Representative synthetic procedures for the **TA-Nap** fluorescent probe were shown in **Supplementary Scheme 2**. Detailed reaction condition information and characterization data were described as follows:

**Synthesis of 6-(dimethylamino)-1*H*, 3*H*-benzo [*de*] isochromene-1, 3-dione** (**Nap**). 4-Bromo-1,8-naphthalic anhydride (554.1 mg, 2.0 mmol), Dimethylamine (40 wt.% solution in water) (680 mL, 6.0 mmol), and CuSO_4_ (63.8 mg, 0.4 mmol) were added to 5 mL of *N*, *N*-Dimethylformamide, and the reaction refluxed for 3 hours. The reaction mixture was cooled to room temperature, poured into water, extracted with Ethyl acetate, collected the organic phase, and dried with anhydrous Na_2_SO_4_. The crude product was eluted with Petroleum ether : Ethyl acetate = 2 : 1 (v/v) to obtain a yellow-brown solid product with a yield of 85%. This product was directly used as a raw material for the synthesis of **Nap-3P-N_3_** without ^1^H-NMR confirmation.

**Synthesis of 2-(2-(2-(2-(2-azidoethoxy) ethoxy) ethoxy) ethyl)-6-(dimethylamino)-1*H*-benzo [*de*] isoquinoline-1, 3(2*H*)-dione** (**Nap-3P-N_3_**). Nap (140.0 mg, 0.58 mmol) and Azide-3PEG-amine (153 μL, 0.70 mmol) were dissolved in 10 mL of Ethanol and refluxed for 3 hours. After the reaction was completed, the ethanol was removed under reduced pressure. The mixture was dissolved in 10 mL of Ethyl acetate, and the organic phase was washed three times with saturated brine. The organic phase was collected and dried over anhydrous Na_2_SO_4_. The mixture was purified by silica gel chromatography (Petroleum ether : Ethyl acetate = 2 : 1, v/v) to obtain a yellow solid (yield: 74%). ^1^H NMR (600 MHz, CDCl_3_) *δ* 8.56 (dt, *J* = 7.3, 1.6 Hz, 1H), 8.49 – 8.42 (m, 2H), 7.69 – 7.63 (m, 1H), 7.12 (dd, *J* = 8.5, 2.9 Hz, 1H), 4.42 (t, *J* = 6.3 Hz, 2H), 3.81 (t, *J* = 6.3 Hz, 2H), 3.70 (dd, *J* = 5.9, 3.6 Hz, 2H), 3.67 – 3.60 (m, 6H), 3.57 (dt, *J* = 6.1, 2.6 Hz, 2H), 3.35 (t, *J* = 5.1 Hz, 2H), 3.11 (d, *J* = 2.2 Hz, 6H).

**Synthesis of (4*S*, 4a*S*, 5a*R*, 6a*R*, 8*R*, 9*S*, 11a*S*, 11b*R*)-9-((*S*)-1-((*R*)-4, 5-dimethyl-6-oxo-3, 6-dihydro-2*H*-pyran-2-yl)ethyl)-8-hydroxy-9, 11b-dimethyl-1-oxo-1, 4, 5a, 6, 6a, 7, 8, 9, 10, 11, 11a, 11b-dodecahydrocyclopenta [1, 2] phenanthro [8a, 9-*b*] oxiren-4-yl 5-(1-(2-(2-(2-(2-(6-(dimethylamino)-1, 3-dioxo-1*H*-benzo [*de*] isoquinolin-2(3*H*)-yl) ethoxy) ethoxy) ethoxy) ethyl)-1*H*-1, 2, 3-triazol-4-yl) pentanoate** (**TA-Nap**). The TA-ester and Nap-3P-N_3_ were used raw materials to obtain the target compound by Click Chemistry reaction. The reaction feeding and work-up steps refer to the synthesis process of **TA-P**. **TA-Nap** was a yellow solid with a yield of 50%. ^1^H NMR (600 MHz, Pyridine-*d_5_*) *δ* 8.70 (dd, *J* = 7.2, 1.2 Hz, 1H), 8.63 (dd, *J* = 8.2, 1.5 Hz, 1H), 8.41 (dd, *J* = 8.5, 1.2 Hz, 1H), 7.82 (s, 1H), 7.64 – 7.61 (m, 1H), 7.26 – 7.23 (m, 1H), 7.08 – 7.04 (m, 1H), 6.74 (d, *J* = 4.9 Hz, 1H), 6.48 (d, *J* = 9.8 Hz, 1H), 5.19 (dt, *J* = 12.9, 3.5 Hz, 1H), 5.10 (d, *J* = 6.2 Hz, 1H), 4.65 (td, *J* = 6.5, 2.4 Hz, 2H), 4.57 (t, *J* = 5.2 Hz, 2H), 4.46 (m, 1H), 4.01 (td, *J* = 6.5, 2.6 Hz, 2H), 3.82 (m, 2H), 3.79 – 3.73 (m, 2H), 3.63 (dt, *J* = 5.4, 3.4 Hz, 2H), 3.57 (dd, *J* = 5.9, 3.3 Hz, 2H), 3.52 (dd, *J* = 5.9, 3.0 Hz, 3H), 2.92 (s, 6H), 2.78 (t, *J* = 7.1 Hz, 2H), 2.56 (dd, *J* = 7.5, 3.5 Hz, 1H), 2.52 – 2.45 (m, 3H), 2.40 – 2.36 (m, 1H), 2.34 (dt, *J* = 6.5, 3.3 Hz, 2H), 2.30 (d, *J* = 2.2 Hz, 1H), 2.28 (s, 1H), 2.04 (s, 1H), 1.89 – 1.85 (m, 3H), 1.76 (s, 3H), 1.74 – 1.69 (m, 3H), 1.67 (s, 3H), 1.40 – 1.35 (m, 2H), 1.29 (s, 1H), 1.27 (d, *J* = 1.6 Hz, 3H), 1.23 (s, 3H), 1.19 (d, *J* = 7.4 Hz, 3H). ^13^C NMR (101 MHz, Pyridine-*d_5_*) *δ* 201.00, 172.25, 166.60, 164.38, 163.76, 156.86, 150.17, 149.09, 147.30, 140.68, 140.17, 135.77, 134.74, 134.28, 133.73, 132.44, 131.35, 130.77, 125.20, 124.85, 123.76, 122.72, 122.22, 121.72, 114.91, 113.33, 81.36, 78.46, 72.18, 70.54, 70.36, 69.54, 68.07, 61.56, 60.26, 52.84, 49.95, 48.13, 44.20, 43.62, 42.01, 41.35, 39.16, 33.58, 32.71, 30.58, 30.27, 29.74, 28.81, 25.42, 24.99, 24.50, 22.98, 19.95, 15.17, 12.35, 11.61.

Representative synthetic procedures for the **TA-DA** photocrosslinkable probe were shown in **Supplementary Scheme 3**. Detailed reaction condition information and characterization data were described as follows:

**Synthesis of (4*S*, 4a*S*, 5a*R*, 6a*R*, 8*R*, 9*S*, 11a*S*, 11b*R*)-9-((*S*)-1-((*R*)-4, 5-dimethyl-6-oxo-3, 6-dihydro-2*H*-pyran-2-yl)ethyl)-8-hydroxy-9,11b-dimethyl-1-oxo-1, 4, 5a, 6, 6a, 7, 8, 9, 10, 11, 11a, 11b-dodecahydrocyclopenta [1, 2] phenanthrol [8a, 9-*b*] oxiren-4-yl 3-(3-methyl-3*H*-diazirin-3-yl) propanoate** (**TA-DA**). The reaction feeding and work-up steps refer to the synthesis process of the **TA-ester**. The difference is using 3-(3-Methyl-3H-diazirin-3-yl) propanoic acid instead of 6-Heptynoic acid during the feeding process. **TA-DA** was a white solid with a yield of 60%. ^1^H NMR (600 MHz, Pyridine-*d_5_*) *δ* 7.25 (dd, *J* = 9.9, 6.2 Hz, 1H), 6.52 (d, *J* = 9.8 Hz, 1H), 5.20 – 5.18 (m, 1H), 5.13 (d, *J* = 6.2 Hz, 1H), 5.09 (d, *J* = 6.2 Hz, 1H), 4.47 (t, *J* = 8.1 Hz, 1H), 3.53 (d, *J* = 2.3 Hz, 1H), 2.57 (dt, *J* = 10.6, 7.2 Hz, 1H), 2.48 (d, *J* = 9.0 Hz, 2H), 2.41 – 2.37 (m, 2H), 2.33 (d, *J* = 4.9 Hz, 1H), 2.32 – 2.30 (m, 1H), 2.09 – 2.02 (m, 1H), 1.93 (s, 1H), 1.88 (d, *J* = 2.1 Hz, 3H), 1.85 – 1.81 (m, 2H), 1.76 (s, 3H), 1.66 (s, 2H), 1.64 (dd, *J* = 7.7, 3.5 Hz, 2H), 1.41 (s, 1H), 1.38 (dd, *J* = 4.5, 2.5 Hz, 1H), 1.36 (d, *J* = 2.8 Hz, 1H), 1.26 (d, *J* = 5.3 Hz, 2H), 1.24 (s, 3H), 1.20 (d, *J* = 7.2 Hz, 3H), 0.89 (s, 3H). ^13^C NMR (101 MHz, Pyridine-*d_5_*) *δ* 200.89, 171.27, 166.60, 140.30, 140.20, 134.25, 133.92, 123.76, 122.72, 121.72, 81.37, 78.46, 72.61, 61.51, 60.32, 52.84, 48.10, 43.61, 42.00, 41.33, 32.73, 30.57, 29.59, 28.50, 25.14, 24.97, 23.99, 22.97, 19.95, 19.07, 15.18, 12.35, 11.62.

**Copies of compounds NMR spectra**

^1^H NMR spectrum of compound **TA-ester**

^1^H NMR spectrum of compound **TA-P**

^13^C NMR spectrum of compound **TA-P**

^1^H NMR spectrum of compound **TA-V**

^13^C NMR spectrum of compound **TA-V**

^1^H NMR spectrum of compound **TA-N**

^13^C NMR spectrum of compound **TA-N**

^1^H NMR spectra of compound **TA-B**

^13^C NMR spectrum of compound **TA-B**

^1^H NMR spectrum of compound **TA-yne**

^1^H NMR spectrum of compound **N_3_-2P-Biotin**

^1^H NMR spectrum of compound **TA-Biotin**

^13^C NMR spectrum of compound **TA-Biotin**

^1^H NMR spectrum of compound **Nap-3P-N_3_**

^1^H NMR spectrum of compound **TA-Nap**

^13^C NMR spectrum of compound **TA-Nap**

^1^H NMR spectrum of compound **TA-DA**

^13^C NMR spectrum of compound **TA-DA**

**References**

[1] Li Y, Duah E, Long N, Persaud A, VanGundy Z, Magliery T, Poi MJ. An efficient and quantitative assay for epitope-tagged therapeutic protein development with a capillary western system. *Bioanalysis*. 2019, 11(6): 471-483.

[2] Li W, Han JL, Entcheva E. Protein and mRNA Quantification in Small Samples of Human-Induced Pluripotent Stem Cell-Derived Cardiomyocytes in 96-Well Microplates. *Methods Mol Biol*. 2022, 2485: 15-37.

[3] Schiattarella GG, Altamirano F, Tong D, French KM, Villalobos E, Kim SY, Luo X, Jiang N, May HI, Wang ZV, Hill TM, Mammen PPA, Huang J, Lee DI, Hahn VS, Sharma K, Kass DA, Lavandero S, Gillette TG, Hill JA. Nitrosative stress drives heart failure with preserved ejection fraction. *Nature*. 2019, 568(7752): 351-356.

[4] Yun SM, Moulaei T, Lim D, Bang JK, Park JE, Shenoy SR, Liu F, Kang YH, Liao C, Soung NK, Lee S, Yoon DY, Lim Y, Lee DH, Otaka A, Appella E, McMahon JB, Nicklaus MC, Burke TR Jr, Yaffe MB, Wlodawer A, Lee KS. Structural and functional analyses of minimal phosphopeptides targeting the polo-box domain of polo-like kinase 1. *Nat Struct Mol Biol*. 2009, 16(8): 876-882.

[5] Śledź P, Stubbs CJ, Lang S, Yang YQ, McKenzie GJ, Venkitaraman AR, Hyvönen M, Abell C. From crystal packing to molecular recognition: prediction and discovery of a binding site on the surface of polo-like kinase 1. *Angew Chem Int Ed Engl*. 2011, 50(17): 4003-4006.

[6] Yang Liu, Mengzhu Zheng, Zhilu Ma, Yirong Zhou, Junfeng Huo, Wenbo Zhang, Yu Liu, Yuanyuan Guo, Xuechen Zhou, Hua Li, Lixia Chen. Design, synthesis, and evaluation of PD-L1 degraders to enhance T cell killing activity against melanoma. *Chin Chem Lett*. 2023, 34(5): 107762.27.
